# Interaction data are identifiable even across long periods of time

**DOI:** 10.1038/s41467-021-27714-6

**Published:** 2022-01-25

**Authors:** Ana-Maria Creţu, Federico Monti, Stefano Marrone, Xiaowen Dong, Michael Bronstein, Yves-Alexandre de Montjoye

**Affiliations:** 1 Department of Computing, Imperial College London, London, SW7 2AZ UK; 2 Data Science Institute, Imperial College London, London, SW7 2AZ UK; 3 Faculty of Informatics, Università della Svizzera Italiana, 6904 Lugano, Switzerland; 4 Twitter, London, W1B 5DL UK; 5 University of Naples Federico II, 80125 Naples, Italy; 6 Department of Engineering Science, University of Oxford, Oxford, OX2 6ED UK

**Keywords:** Computational science, Society

## Abstract

Fine-grained records of people’s interactions, both offline and online, are collected at large scale. These data contain sensitive information about whom we meet, talk to, and when. We demonstrate here how people’s interaction behavior is stable over long periods of time and can be used to identify individuals in anonymous datasets. Our attack learns the profile of an individual using geometric deep learning and triplet loss optimization. In a mobile phone metadata dataset of more than 40k people, it correctly identifies 52% of individuals based on their 2-hop interaction graph. We further show that the profiles learned by our method are stable over time and that 24% of people are still identifiable after 20 weeks. Our results suggest that people with well-balanced interaction graphs are more identifiable. Applying our attack to Bluetooth close-proximity networks, we show that even 1-hop interaction graphs are enough to identify people more than 26% of the time. Our results provide strong evidence that disconnected and even re-pseudonymized interaction data can be linked together making them personal data under the European Union’s General Data Protection Regulation.

## Abstract (web summary)

Large amounts of interaction data are collected by messaging apps, mobile phone carriers, and social media. Creţu et al. propose a behavioral profiling attack model and show that the stability of people’s interaction networks over time can allow individuals to be re-identified in interaction datasets.

## Introduction

An increasing fraction of our online and offline interactions are now captured by technology^1^. Large amounts of interaction data are now collected by messaging apps, mobile phone carriers, social media companies, and other apps to operate their service or for research purposes. Interaction data typically consist of the pseudonyms of the interaction parties, the timestamp of the interaction, and possibly further information. Mobile phone interaction data have been used to study the linguistic divide in a country^2^, to study the interaction patterns of individuals with close connections over time^3^, or to forecast the spatial spread of epidemics^4^. Similarly, interaction data have been used to study the spread of misinformation on Twitter^5,6^, the characteristics of news retweet networks during elections^7^, or the effect of Facebook friendship ties in political mobilization^8^. Finally, close-proximity interaction data have been collected using Bluetooth to study human behavior^9–11^ and are currently at the core of COVID-19 contact tracing apps aiming to help control the spread of the disease.

Despite previous claims^12,13^, interaction data are deeply personal and sensitive. They record with high precision who we talk to or meet, at what time, and for how long. Sensitive information can furthermore often be inferred from interaction data. Previous research, for instance, showed how algorithms can predict who a person’s significant other is^14^, their wealth^15,16^, demographics^17,18^, the propensity to overspend^19^, personality traits^20^, and other attributes^21^ from interaction data. Some works even leveraged homophily or network ties when making predictions^22^. Legal scholars and privacy advocates have long argued that interaction data are as sensitive as the content of the communication and that “metadata are data”^23,24^. Mobile phone metadata have been at the core of the Snowden revelations and their collection was later deemed illegal in ACLU vs. Clapper^25,26^. More recently, the proportionality of contact tracing apps developed in the context of the COVID-19 pandemic has been questioned^27–29^.

Interaction data can be shared or sold to third parties without users’ consent, so long as they are anonymized. According to current data protection regulations such as the European Union’s General Data Protection Regulation (GDPR)^30^, or the California Consumer Privacy Act (CCPA), anonymized (or de-identified) data are no longer considered as personal data. The European Data Protection Board (EDPB) predecessor, the Article 29 Working Party, defined anonymization as resistance to singling out, linkability, and inference attacks^31^. In particular, the linkability criterion refers to “the ability to link, at least, two records concerning the same data subject.” While guidances are subject to the interpretation of the courts, matching identities between two pseudonymous datasets would likely mean that they are not anonymous under GDPR. Both legislations emphasize that personal data should not be stored for longer than necessary and then deleted or anonymized, with terms of service suggesting the latter to be common practice^32–34^.

Matching attacks have long been used to identify individuals in datasets using matching auxiliary information, calling into question their anonymity. In one seminal study, zip code, birth date, and gender were used to identify the Governor of Massachusetts William Weld^35^; in another, the movies people had watched were used^36^. In 2013, it was shown that four points, approximate places and times, were enough to uniquely identify someone in location data 95% of the time^37^, with formal similarity measures being proposed for approximate matching^38^. Numerous matching attacks have been proposed for interaction and graph data, both using exact^39–46^ or approximate^47–54^ matching information. Graph matching^55–58^ and anchor links prediction^59,60^ are two closely related problems.

We here propose a profiling attack for interaction data based on geometric deep learning^61^. While matching attacks rely on auxiliary information fairly stable over time (gender, zip code, etc.) or from the same time period (spatio-temporal points, movies watched, etc.), profiling attacks use auxiliary information from one time period to profile and identify a person in another non-overlapping time period. This makes them more broadly applicable, as the auxiliary data does not have to come from the same time period as the dataset.

Using a graph attention neural network^62^, we learn an individual’s behavioral profile by building a vector representation (embedding) of their *weekly* *k*-hop interaction network. Our weekly profiles use only behavioral features, aggregating both node features and topological information typically present in interaction data, and are optimized for identification. In a mobile phone dataset of more than 40k people, our model was able to correctly identify a person 52% of the time based on their 2-hop interaction network (*k* = 2). Using only a person’s interactions with their direct contacts (*k* = 1), our model could still identify them 15% of the time. We further show that the accuracy of our model only decreases slowly as time passes with 24% of the people still being correctly identified after 20 weeks (*k* = 2), thus making identification a real risk in practice. Finally, we show that our general graph profiling approach can be applied to other types of interaction data. We apply our model to Bluetooth close-proximity data similar to the one collected by COVID-19 contact tracing apps for more than 500 people and show that it is able to link together 1-hop interaction networks with 26% accuracy. Our results provide evidence that disconnected and even re-pseudonymized interaction data remain identifiable even across long periods of time. These results strongly suggest that current practices may not satisfy the anonymization standard set forth by the EDPB in particular with regard to the linkability criteria.

## Results

### Setup

Our attack exploits the stability over time of people’s interaction patterns to identify individuals in a dataset of interactions using auxiliary *k*-hop interaction data from a disjoint time period.

**Fig. 1:**
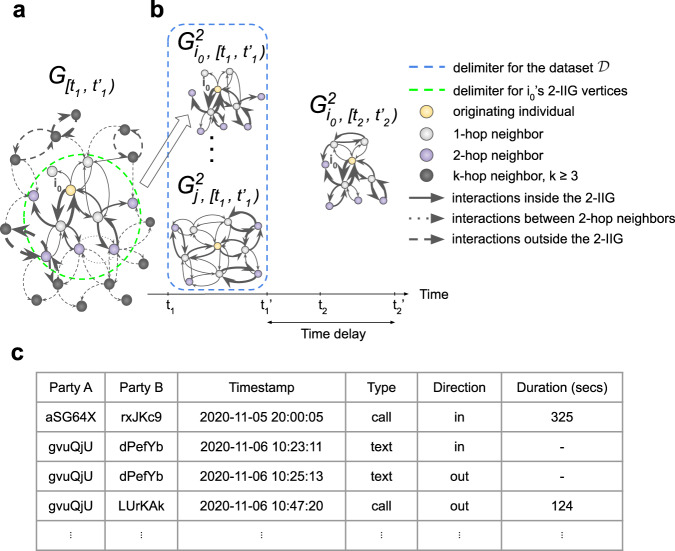
Setup of the behavioral profiling attack. **a** An example of a 2-IIG is highlighted in the larger graph it comes from. The vertices of the 2-IIG (inside the dashed green circle) are respectively the originating individual (in yellow), 1-hop neighbors (in gray), and the 2-hop neighbors (in purple). In solid lines are the edges that are part of the 2-IIG: all the edges between the originating and 1-hop neighbors; between the 1-hop neighbors; and between 1-hop and 2-hop neighbors, but excluding those between 2-hop neighbors (dotted lines). Dashed lines are all the other edges. For simplicity, all edges are shown as a single directed edge of thickness proportional to the total number of interactions. **b** The data available to the attacker consist of (left) 2-IIGs coming from the time period $[{t}_{1},{t}_{1}^{\prime})$, usually as part of an anonymized dataset, and (right) auxiliary 2-IIG data about a target individual *A* (${G}_{{i}_{0},[{t}_{2},{t}_{2}^{\prime})}^{2}$). While we here display auxiliary data coming from a later period in time, our attack applies equally to cases where the auxiliary data comes from an earlier time period. **c** An example of mobile phone interaction data. Each interaction contains the pseudonyms of the parties A and B, timestamp, type of interactions, direction (equal to “out” if A initiated it, “in” otherwise), and the duration for calls. In this example, the person identified by “gvuQjU” received a text from another person, identified by “dPefYb'', to whom the former responded 2 min later. After 22 min, “gvuQjU” called another individual, identified by “LUrKAk'', for a duration of 124 s.

We consider a service *S* collecting data about the interactions it is mediating. We denote by ${{{{{{{\mathcal{I}}}}}}}}$ the set of individuals taking part in the communications recorded by *S*. For example, ${{{{{{{\mathcal{I}}}}}}}}$ could be the set of users of a contact tracing or messaging application or the subscribers of a mobile phone carrier and their contacts. We call interaction data the record describing the interaction between two individuals using *S*, consisting of the pseudonym of the two individuals, a timestamp, and sometimes other information. We define a time period ${{{{{{{\mathcal{T}}}}}}}}=[t,t^{\prime} )$ as the set of all timestamps between a start *t* (inclusive) and end $t^{\prime}$ (exclusive). Given a time period ${{{{{{{\mathcal{T}}}}}}}}$, we define the interaction graph ${G}_{{{{{{{{\mathcal{T}}}}}}}}}$ as the directed multigraph with node set ${{{{{{{\mathcal{I}}}}}}}}$ and an edge between two nodes for each interaction between the corresponding individuals at a timestamp in the time period ${{{{{{{\mathcal{T}}}}}}}}$. Each edge is endowed with additional data *m* describing the interaction. For example, if *S* is a mobile operator, *m* would be the timestamp, the type of interaction (i.e., call or text), its direction (i.e., which party initiated it), and the duration for calls (see Fig. 1). If *S* is a close-proximity app, *m* would be the timestamp and the strength of the signal. We denote by *k*-hop neighbor of a node $v\in {{{{{{{\mathcal{I}}}}}}}}$ any node $w\in {{{{{{{\mathcal{I}}}}}}}}$ such that the shortest path between *v* and *w* in ${G}_{{{{{{{{\mathcal{T}}}}}}}}}$ is of length *k*. Given a time period ${{{{{{{\mathcal{T}}}}}}}}$, $i\in {{{{{{{\mathcal{I}}}}}}}}$ an individual and *k* = 1, 2, …, we define the *k*-hop Individual Interaction Graph (*k*-IIG) ${G}_{i,{{{{{{{\mathcal{T}}}}}}}}}^{k}$ as the subgraph induced in ${G}_{{{{{{{{\mathcal{T}}}}}}}}}$ by the set of nodes situated on paths of length at most *k* starting at node *i*, excluding interactions between the *k*-hop neighbors themselves. We denote by *i* the originating individual of *k*-IIG ${G}_{i,{{{{{{{\mathcal{T}}}}}}}}}^{k}$. Figure 1 shows an example of a 2-IIG.

Our attack model assumes (see Fig. 1) that a malicious agent, the attacker, has access to (1) a dataset ${{{{{{{\mathcal{D}}}}}}}}=\{{G}_{i,\left[{t}_{1},{t}_{1}^{\prime}\right)}^{k}\ \ :\ \ i\in {{{{{{{\mathcal{I}}}}}}}}^{\prime} \}$ consisting of the *k*-IIGs of people in ${{{{{{{\mathcal{I}}}}}}}}^{\prime} \subset {{{{{{{\mathcal{I}}}}}}}}$ from time period ${{{{{{{{\mathcal{T}}}}}}}}}_{1}=\left[{t}_{1},{t}_{1}^{\prime}\right)$, as well as to (2) auxiliary data ${G}_{{i}_{0},\left[{t}_{2},{t}_{2}^{\prime}\right)}^{k}$ consisting in the *k*-IIG of a known target individual ${i}_{0}\in {{{{{{{\mathcal{I}}}}}}}}^{\prime}$, coming from a disjoint time period ${{{{{{{{\mathcal{T}}}}}}}}}_{2}=[{t}_{2},{t}_{2}^{\prime})$ (i.e., ${t}_{1}^{\prime}\ \le \ {t}_{2}$ or ${t}_{2}^{\prime}\ \le \ {t}_{1}$). We further assume that the attacker knows, for each *k*-IIG, which node is at the center of the *k*-IIG (originating node), and that the *k*-IIGs are pseudonymized, meaning that a node will have a different pseudonym in each graph it appears in. The attacker’s goal is to find the target *i*_0_ in ${{{{{{{\mathcal{D}}}}}}}}$, i.e., find the ${G}_{i,[{t}_{1},{t}_{1}^{\prime})}^{k}\in {{{{{{{\mathcal{D}}}}}}}}$ such that *i* = *i*_0_. If successful, the attacker is said to have identified *i*_0_ and is able to retrieve all their interactions from time period $[{t}_{1},{t}_{1}^{\prime})$. We denote by time delay the quantity $D={t}_{2}^{\prime}-{t}_{1}^{\prime}$. We refer the reader to the section “Discussion” for examples.

### Model

Our *k*-IIG-based Behavioral Profiling approach (BP-IIG) first computes a time-dependent profile of an individual in the form of a vector representation (embedding). We apply a neural network to people’s *k*-IIGs before identifying them using the nearest neighbor in the embedding space.

One of the key challenges for using deep learning in such a setting is that, unlike images or acoustic signals, graphs have a non-Euclidean structure. Recently, generalizations of deep learning architectures (in particular, convolutional neural networks) have been proposed for graph-structured data^61,63–65^, with successful applications to biology^66–71^, medicine^72^, and social network analysis^6,66^.

To compute the time-dependent profile embedding of individual *i*, we aggregate the interaction data from their *k*-IIG ${G}_{i,{{{{{{{\mathcal{T}}}}}}}}}^{k}$, using the nodes’ bandicoot features^73^ (see Supplementary Tables 1 and 2 and the Supplementary Methods) and by employing a multi-layer graph neural network (*k* ≥ 2, see the “Methods” section) of the form:

1 $${{{{{{{{\bf{h}}}}}}}}}_{i}^{(s)}={\xi }^{(s)}\left(\left[{{{{{{{{\bf{h}}}}}}}}}_{i}^{(s-1)},\mathop{\sum}\limits_{j\in {{{{{{{\mathcal{N}}}}}}}}(i)}{\alpha }_{j}^{(s)}{{{{{{{{\bf{h}}}}}}}}}_{j}^{(s-1)}\right]\right)$$

2 $${\alpha }_{j}^{(s)}=\frac{{\alpha }^{(s)}({{{{{{{{\bf{h}}}}}}}}}_{i}^{(s-1)},{{{{{{{{\bf{h}}}}}}}}}_{j}^{(s-1)})}{\mathop{\sum}\limits_{l\in {{{{{{{\mathcal{N}}}}}}}}(i)}{\alpha }^{(s)}({{{{{{{{\bf{h}}}}}}}}}_{i}^{(s-1)},{{{{{{{{\bf{h}}}}}}}}}_{l}^{(s-1)})}$$

where the output ${{{{{{{{\bf{h}}}}}}}}}_{i}^{(s-1)}$ of layer *s*−1 is passed as the input to layer *s* = 1, …, *S*. For each layer 1 ≤ *s* ≤ *S*, *ξ*^(*s*)^ is a non-linear parametric function implemented as a multi-layer perceptron (MLP) with one hidden layer, followed by ${{\mathbb{L}}}_{2}$-normalization. Finally, *α*^(*s*)^ denotes the attention weight computed as a nonlinear parametrized function of the features of node *i* and its neighbor $j\in {{{{{{{\mathcal{N}}}}}}}}(i)$. The neural attention mechanism, previously shown to improve performance in tasks such as object recognition^74^ and machine translation^75^, has been adapted for graph inputs by aggregating a node’s neighborhood features via a weighted average over the features of the neighbors^62^. The attention weights are potentially different for distinct neighbors and are optimized for a specific learning task.

The network is applied to the input node-wise features ${{{{{{{{\bf{h}}}}}}}}}_{i}^{(0)}$ and its output ${{{{{{{{\bf{h}}}}}}}}}_{i}^{(S)}={{{{{{{\bf{h}}}}}}}}({G}_{i,{{{{{{{\mathcal{T}}}}}}}}}^{k};{{{{{{{\boldsymbol{\Theta }}}}}}}})$ is used as the embedding of individual *i*, with **Θ** denoting the network parameters of *ξ*^(*s*)^ and *α*^(*s*)^ optimized during training.

The neural network is trained to optimize the matching accuracy, using the triplet loss^76^, which optimizes the profile embeddings of the same individual at different time periods (positive pair) to be closer to each other than to those of different individuals at any time period (negative pair). A triplet of *k*-IIGs $({G}_{i,{{{{{{{\mathcal{T}}}}}}}}}^{k},{G}_{i,{{{{{{{\mathcal{T}}}}}}}}^{\prime} }^{k},{G}_{i^{\prime} ,{{{{{{{\mathcal{T}}}}}}}}^{\prime\prime} }^{k})$ contains data from two individuals $i\,\ne\, i^{\prime}$, such that there are two *k*-IIGs from *i*, coming from time periods that are not equal, but could be overlapping ${{{{{{{\mathcal{T}}}}}}}}\ne \,{{{{{{{\mathcal{T}}}}}}}}^{\prime}$, and a *k*-IIG from $i^{\prime}$ from a time period ${{{{{{{\mathcal{T}}}}}}}}^{\prime\prime}$ (not necessarily different from ${{{{{{{\mathcal{T}}}}}}}}$ or ${{{{{{{\mathcal{T}}}}}}}}^{\prime}$). Let ${{{{{{{\bf{h}}}}}}}}({{{{{{{\boldsymbol{\Theta }}}}}}}})={{{{{{{\bf{h}}}}}}}}({G}_{i,{{{{{{{\mathcal{T}}}}}}}}}^{k};{{{{{{{\boldsymbol{\Theta }}}}}}}})$, ${{{{{{{{\bf{h}}}}}}}}}^{+}({{{{{{{\boldsymbol{\Theta }}}}}}}})={{{{{{{\bf{h}}}}}}}}({G}_{i,{{{{{{{\mathcal{T}}}}}}}}^{\prime} }^{k};{{{{{{{\boldsymbol{\Theta }}}}}}}})$ and ${{{{{{{{\bf{h}}}}}}}}}^{-}({{{{{{{\boldsymbol{\Theta }}}}}}}})={{{{{{{\bf{h}}}}}}}}({G}_{i^{\prime} ,{{{{{{{\mathcal{T}}}}}}}}^{\prime\prime} }^{k};{{{{{{{\boldsymbol{\Theta }}}}}}}})$ denote the respective embeddings. The triplet loss

3 $$\ell ({{{{{{{\boldsymbol{\Theta }}}}}}}})=\max (0,\parallel \!{{{{{{{\bf{h}}}}}}}}({{{{{{{\boldsymbol{\Theta }}}}}}}})-{{{{{{{{\bf{h}}}}}}}}}^{+}({{{{{{{\boldsymbol{\Theta }}}}}}}}){\parallel }_{2}-\parallel \!{{{{{{{\bf{h}}}}}}}}({{{{{{{\boldsymbol{\Theta }}}}}}}})-{{{{{{{{\bf{h}}}}}}}}}^{-}({{{{{{{\boldsymbol{\Theta }}}}}}}}){\parallel }_{2}+\lambda )$$

tries to ensure that the profiles (**h**, **h**^+^) of the positive pair (i.e., the pair of profiles constructed from interaction data of the same individual, but different time periods) are closer than those (**h**, **h**^−^) of the negative pair (i.e., the pair of profiles constructed from *i* and another individual’s interaction data in possibly, but not necessarily, different time periods ${{{{{{{\mathcal{T}}}}}}}}$ and ${{{{{{{\mathcal{T}}}}}}}}^{\prime\prime}$) by at least a margin *λ*. We average the triplet loss over a training set of positive and negative pairs and minimize it w.r.t. the network parameters **Θ**. The optimal parameters Θ^*^ obtained as the result of training are then used for the attack.

The attacker trains the embedding network on data from the dataset ${{{{{{{\mathcal{D}}}}}}}}$ (see the “Methods” section). To identify the target individual *i*_0_ in ${{{{{{{\mathcal{I}}}}}}}}^{\prime}$, the attacker computes the Euclidean distance ${d}_{{i}_{0},j}=\,\parallel \!\!{{{{{{{\bf{h}}}}}}}}({G}_{{i}_{0},{{{{{{{{\mathcal{T}}}}}}}}}_{2}^{\prime}}^{k};{{{{{{{{\boldsymbol{\Theta }}}}}}}}}^{* })-{{{{{{{\bf{h}}}}}}}}({G}_{j,{{{{{{{{\mathcal{T}}}}}}}}}_{1}^{\prime}}^{k};{{{{{{{{\boldsymbol{\Theta }}}}}}}}}^{* }){\parallel }_{2}$ between the profile of *i*_0_ from target time period ${{{{{{{{\mathcal{T}}}}}}}}}_{2}^{\prime}\subset {{{{{{{{\mathcal{T}}}}}}}}}_{2}$ and the profiles of all the individuals $j\in {{{{{{{\mathcal{D}}}}}}}}$ from a reference time period ${{{{{{{{\mathcal{T}}}}}}}}}_{1}^{\prime}\subset {{{{{{{{\mathcal{T}}}}}}}}}_{1}$ of same length as ${{{{{{{{\mathcal{T}}}}}}}}}_{2}^{\prime}$. If the candidate with the smallest distance is (resp. *R* candidates with the smallest distance contains) the target individual (i.e., *i*_0_ ∈ {*j*_1_, …, *j*_*R*_}), we say that we have correctly identified *i* (resp. within rank *R*).

### Mobile phone interaction data

We use a mobile phone interaction dataset composed of the 3-IIGs of *N* = 43, 606 subscribers of a mobile carrier collected over a period of *T* = 35 consecutive weeks ${{{{{{{\mathcal{T}}}}}}}}={{{{{{{{\mathcal{W}}}}}}}}}_{1}\cup \ldots \cup {{{{{{{{\mathcal{W}}}}}}}}}_{T}:={{{{{{{{\mathcal{W}}}}}}}}}_{1:T}$, where ${{{{{{{{\mathcal{W}}}}}}}}}_{n}=[{t}_{n},{t}_{n+1})$ denotes the *n*th week, with 1 ≤ *n* ≤ *T* and *t*_*n*+1_ and *t*_*n*_ differing by one week. The interaction data contain the pseudonyms of the interacting parties, timestamp, as well as the type of interaction (call or text), the direction of the interaction, and the duration of calls. We here consider the auxiliary profiling information available to the attacker to be the *k*-IIG of the target individual from a week ${{{{{{{{\mathcal{T}}}}}}}}}_{2}\in \{{{{{{{{{\mathcal{W}}}}}}}}}_{T^{\prime} +1},\ldots ,{{{{{{{{\mathcal{W}}}}}}}}}_{T}\}$ and the anonymous dataset to be the *k*-IIGs of all the *N* people from the first $T^{\prime} =15$ weeks of data (${{{{{{{{\mathcal{T}}}}}}}}}_{1}={{{{{{{{\mathcal{W}}}}}}}}}_{1:T^{\prime} }$). We report the probability of identification within rank *R*, defined as the fraction of people among the *N* subscribers who are correctly identified within rank *R* (averaged over 10 runs).

**Fig. 2:**
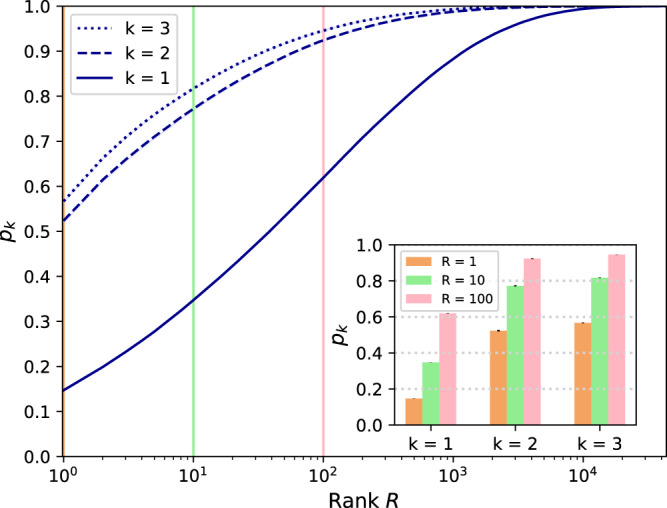
Probability of identification. For each *k* ∈ {1, 2, 3}, we plot *p*_*k*_, the probability of identification within rank *R* ∈ {1, …, 43, 606} when the time delay is *D* = 1 week, with the 95% confidence interval shown in light blue. (Inset) shows the probability of identification for ranks 1, 10, and 100, with error bars for the 95% confidence interval. Our model correctly identifies people 52.4% of the time for *k* = 2. The probability of correct identification is still high at *p*_*k*=1_ = 14.7% for *k* = 1 and slightly increases *p*_*k*=3_ = 56.7% when *k* increases from 2 to 3. Our model ranks the correct candidate among the top 10 predictions *p*_*k*=2_ = 77.2% of the time and among the top 100 predictions *p*_*k*=2_ = 92.4% of the time for *k* = 2.

Figure 2 shows that our model correctly identifies people *p*_*k*=2_ = 52.4% of the time in a dataset of 43.6k people with *k* = 2 i.e. when the attacker has access to an individual’s interactions as well as the interactions of their contacts here with a time delay of a week. It also shows the probability *p* of identification of a target individual within the top *R* matches. Our model is able to rank the correct person among the top 10 candidates *p*_*k*=2_ = 77.2% of the time and among the top 100 candidates, *p*_*k*=2_ = 92.4% of the time.

When *k* = 1, i.e., when the attacker has only access to the individual’s direct interactions, our model is still able to identify people *p*_*k*=1_ = 14.7% of the time. While having access to the 2-hop information helps, our model still performs much better than random for *k* = 1. The probability of identifying the correct person among the top 10 candidates (rank 10) is *p*_*k*=1_ = 34.7% while the rank 100 probability is *p*_*k*=1_ = 61.9%, respectively. Interestingly, having access to information beyond the target’s direct contacts (*k* = 3) only marginally increases the probability of correct identification *p*_*k*=3_ = 56.7% (a 7.9% increase w.r.t. *k* = 2). Higher ranks probabilities similarly increase to *p*_*k*=3_ = 81.7% and *p*_*k*=3_ = 94.6%, respectively, a 5.8% and a 2.4% increase. On the one hand, this marginal increase could be due to the fairly large number of nodes reached with *k* = 3 (121.5 ± 48.8 for *k* = 3 vs. 17.3 ± 13.4 for *k* = 2) thereby limiting the usefulness of data from larger *k* (see Supplementary Note 1). On the other hand, this could also be due to our particular choice of architecture. In particular, while we downsampled the simplified *k*-IIG to contain no more than *τ* = 200 nodes for *k* = 3 (see the Supplementary Methods), the graph neural network architecture might still suffer from over smoothing. Given that new architectures could be developed to leverage information coming from the 3-IIG specifically, from a privacy perspective, our results are thus only a lower bound on the risk of re-identification.

**Fig. 3:**
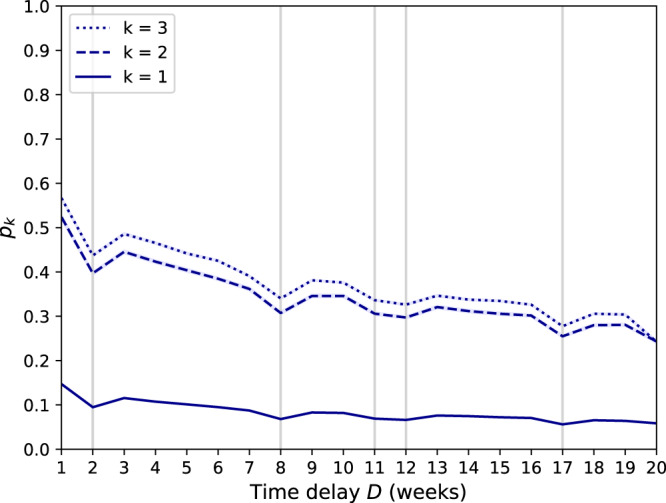
Probability of identification when the time delay increases. We plot *p*_*k*_, the probability of identification within rank 1 for *k* ∈ {1, 2, 3} when the time delay between the dataset and the attacker’s auxiliary information is equal to *D* weeks. The auxiliary information is one week long. The 95% confidence interval is shown in light blue. The vertical gray lines correspond to holidays. While *p*_*k*_ decreases slowly with the time delay, our model correctly identifies people *p*_*k*=2_ = 24.3% of the time even after 20 weeks (*k* = 2). Even for *k* = 1, the probability after 20 weeks is as high as *p*_*k*=1_ = 5.8%. The probability of identification decreases as *y* = *p*_*k*_(*D* = 1)−*α*_*k*_ × (*D*−1), with *α*_1_ = −0.006, *α*_2_ = −0.017 and *α*_3_ = −0.018.

The accuracy of our model is likely to decrease as time passes: people change behavior, make new friends, and lose contact with others. Figure 3 shows that, despite this, the probability of correct identification only slowly decreases with the time delay $D={t}_{2}^{\prime}-{t}_{1}^{\prime}$ (see the section “Setup”). Even after 20 weeks, our model still correctly identifies people *p*_*k*=2_ = 24.3% of the time when *k* = 2. This suggests that the profiles our model extracts from the data capture key behavioral features of individuals. The probability of identification decreases similarly slowly with time for *k* = 3 and *k* = 1.

Interestingly, Fig. 3 shows that the probability of identification (*p*_*k*_) visibly decreases when the time delay is 8, 11, 12, and 17 weeks, respectively. In a post-hoc analysis, we found that they all correspond to weeks containing a national holiday. This further suggests that our model captures a person’s routine weekly behavior, both weekdays and weekends, and consequently loses some accuracy when a user’s behavior changes in response to external events.

We have so far assumed that the attacker has access to a week of a target individual’s data, i.e., their auxiliary information is the target individual’s *k*-IIG from one week. In practice, an attacker might often have access to more weeks of data from an individual. In the D4D challenge, data were for instance re-pseudonymized every 2 weeks^77^ while a company wanting to archive transactional data might decide to pseudonymize and archive it on a monthly basis. To simply evaluate the extent to which more auxiliary data increase accuracy, we combine the predictions from growing sequences of target weeks used as auxiliary data. For $1\ \le \ L\ \le \ T-T^{\prime}$ (*L* denotes the number of weeks in the auxiliary data or ${{{{{{{{\mathcal{T}}}}}}}}}_{2}$), we combine the predictions from the $T^{\prime} +1,\ldots ,(T^{\prime} +L)$th target weeks using a majority vote: the candidate that was ranked first most of the time is the final prediction. The tie-breaks are decided by the lowest total distance between the target individual and the highest-ranked candidate (see Supplementary Note 2).

**Fig. 4:**
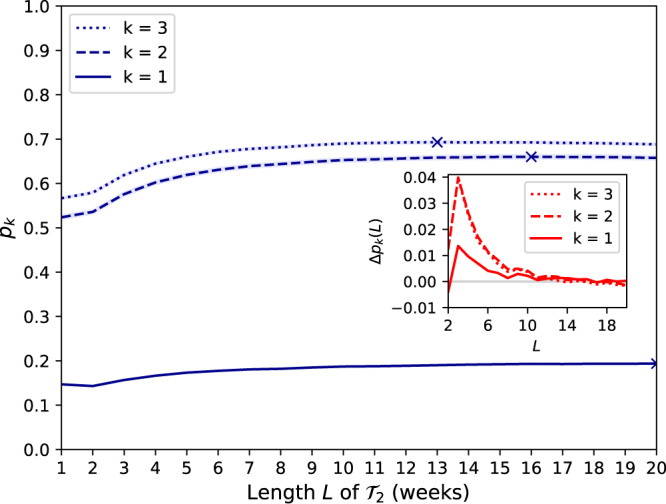
Probability of identification for increasing time period length of auxiliary data. For each *k* ∈ {1, 2, 3}, we plot *p*_*k*_, the probability of correct identification (*R* = 1) when the attacker’s auxiliary data ${{{{{{{{\mathcal{T}}}}}}}}}_{2}$ consist of *L* weeks, 1 ≤ *L* ≤ 20 (the largest value for each *k* is marked). The 95% confidence interval is shown in light blue. (Inset) shows the difference quotient Δ*p*_*k*_(*L*) = *p*_*k*_(*L*) − *p*_*k*_(*L* − 1) for 2 ≤ *L* ≤ 20. The probability of correct identification increases fast before plateauing around *L* = 8 weeks for all values of *k*, even slightly decreasing after *L* = 16 and *L* = 15 for *k* = 2 and *k* = 3, respectively. The largest values are *p*_*k*=1_ = 19.4% at *L* = 20, *p*_*k*=2_ = 66.0% at *L* = 16, and *p*_*k*=3_ = 69.3% at *L* = 13. This shows that having more auxiliary data further improves the performance of the attack, although data that are more distant in time seem less useful than closer ones, even slightly detrimental.

Figure 4 shows how having auxiliary data over several weeks further improves the performance of the attack. For *k* = 2, the probability of correct identification increases from *p*_*k*=2_ = 52.4% with one week of auxiliary data to *p*_*k*=2_ = 66.0% with *L* = 16 weeks. Interestingly, the probability of correct identification for all values of *k* increases fast and then plateaus around *L* = 8, even slightly decreasing after *L* = 16 and *L* = 15 for *k* = 2 and *k* = 3, respectively. Despite having access to more data, the attack is less accurate for increasing time delay. While this might seem surprising at first, we hypothesize this to be due to small changes in people’s behavior over time. This makes auxiliary data that are more distant in time less useful than closer ones and sometimes slightly detrimental. The maximum probability for *k* = 2 is at *L* = 16 weeks (*p*_*k*=2_ = 66.0%) and for *k* = 1 and *k* = 3 at *L* = 20 (*p*_*k*=1_ = 19.4%) and *L* = 13 (*p*_*k*=3_ = 69.3%), respectively. Finally, we show that the accuracy of our attack only decreases slowly with the size of the dataset size (see Supplementary Note 3 and Supplementary Fig. 3).

**Fig. 5:**
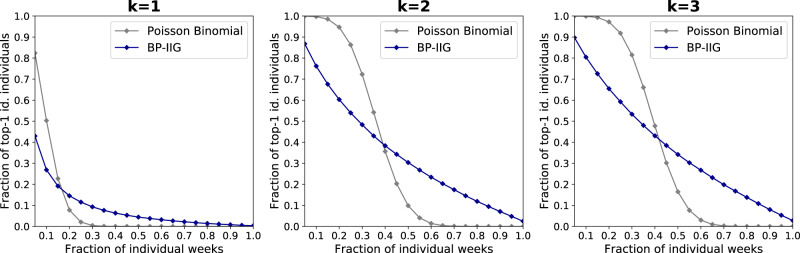
Fraction of identified people vs. fraction of individual weeks. For each *k* ∈ {1, 2, 3}, we plot the fraction of people that are identified in at least a given fraction of individual weeks, using our model (in blue) and according to a Poisson binomial distribution (in gray, averaged over 100 trials). Our attack identifies 38.4% (resp. 14.5% and 38.5%) of the people more often than expected for *k* = 2 (resp. for *k* = 1 and *k* = 3).

We finally perform a post-hoc analysis to better understand who are the people that our model identifies correctly. Figure 5 shows (in blue) in how many weeks a person is correctly identified by our attack, each time using a single week of auxiliary data target weeks (weeks $T^{\prime} +1,\ldots ,T$ of the mobile phone dataset). For instance, for *k* = 2, 86.8% of people are correctly identified by our model at least once (5% of the 20 target weeks). We compare this with a naïve model in which individuals are identified independently in each week with the same probability as our attack, and independently from one another. In the latter setting, the number of weeks when a person is correctly identified follows a Poisson binomial distribution defined as the probability distribution of $B:=\mathop{\sum }\nolimits_{l=T^{\prime} +1}^{T}{B}_{l}$ with *B*_*l*_ ~ Bernoulli(*p*_*l*_), where *p*_*l*_ denotes the probability of identification in target week *l* using our attack (see the Supplementary Note 4). We can see that our attack identifies some people in many more weeks than expected. For *k* = 2, the people we identify more often than expected are correctly identified in at least 40% of the weeks. The two curves cross one another at 20% and 45% for *k* = 1 and *k* = 3 respectively. In all the other initializations of our attack and every *k* ∈ {1, 2, 3}, the lowest abscissa value where our approach outperforms the baseline is the same.

**Fig. 6:**
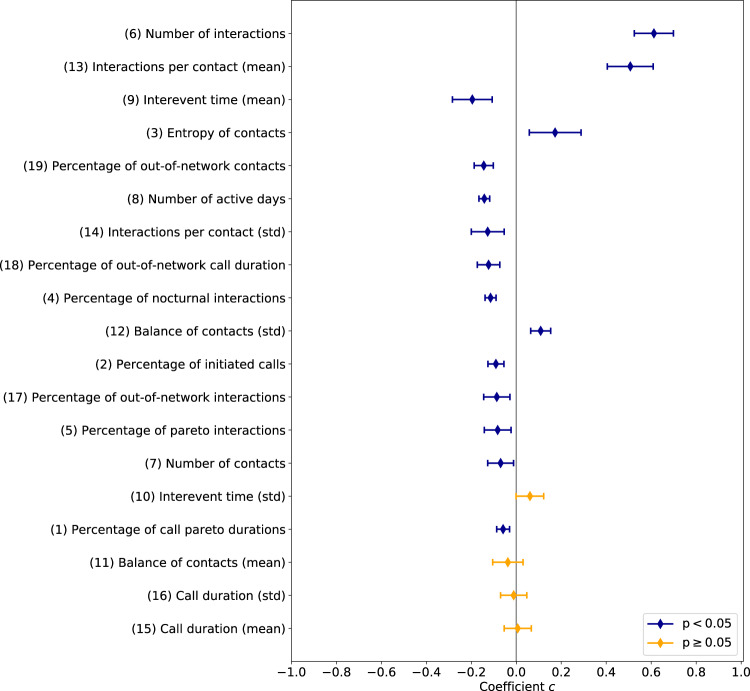
Coefficients of logistic regression for individual identifiability. For each feature, we plot the coefficient *c* (with the 95% confidence interval) of a logistic regression classifier with whether a person is more or less identifiable than expected as the dependent variable. Features are ordered decreasingly from top to bottom according to the absolute value of *c*. When holding all other features constant, these results suggest that having more interactions and a well-balanced interaction graph makes individuals more identifiable.

Figure 6 suggests that, when holding all other features constant, individuals with more interactions, or a well-balanced interaction graph are more identifiable. Using logistic regression, we study in a post-hoc analysis what distinguishes the people our model identifies more often and less often than expected for *k* = 2. Supplementary Table 3 shows the bandicoot features used in this analysis. The largest coefficients (in absolute value), both as individual predictors (see Supplementary Fig. 4) and taken together, are the number of interactions, the mean number of interactions per contact (*c* > 0), and the mean interevent time, (i.e., time elapsed between consecutive interactions) (*c* < 0). Interestingly, a person’s call duration (both mean and standard deviation) seems to have no impact (*p* ≥ 0.05) on identifiability. While the standard deviations of all summary distributions are highly correlated with their mean (*ρ* > 0.7, see Supplementary Fig. 5), they can still be informative even when other features are accounted for, e.g., the standard deviation of the number of interactions per contact. Last, we note that all other features being the same, the lower a person’s number of active days, the more likely they are to be identified, with similar findings for the percentage of nocturnal or out-of-network activity. A more detailed analysis of the logistic regression results and pairwise feature correlations is provided in Supplementary Note 4. While our findings suggest the possible influence of the various behavioral features on identification, a causal analysis is beyond the scope of this paper.

### Bluetooth close-proximity data

To prevent the spread of COVID-19, governments and companies around the world have been developing and releasing a number of contact tracing apps. Contact tracing apps use Bluetooth to collect close-proximity data between users. If a user becomes infected, they upload to a server data allowing their contacts to be informed that they might have been infected. In the centralized model, application users typically upload the temporary pseudonyms of their contacts^78,79^. In the decentralized model, they upload data about themselves, typically cryptographic keys, which their contacts can use to deduce that they might have been infected^80–83^. In another (“hybrid”) system, users upload their encounter keys (corresponding to a pair of user identifiers) instead^84^. Numerous application designs based on these protocols have been proposed and are under active development.

Our attack is, to the best of our knowledge, the first to show how mitigation strategies relying on changing pseudonyms of both the person and of all of their contacts could fail to adequately protect people’s privacy. While it does not target a specific application, protocol, or type of protocol (centralized, decentralized, or hybrid), it could form an effective basis for an attack against any system where an attacker has access to a user’s social graph over two or more time periods. This could be by design in a centralized system (e.g., the UK’s NHSX app reportedly plans to change keys every 24 h^78^) or the results of extra data collection in a decentralized system (e.g., the Belgian system reportedly collects the number of encounters with infected users and, for each encounter, the number of days elapsed since the reported contamination of the other user^85^). While the specifications for the reporting of data for epidemiological purposes are currently under discussion, they are likely to include part or all of the infected user’s social graph.

We evaluate the effectiveness of our attack using a real-world Bluetooth close-proximity network of 587 university students over 4 weeks^11^. Our interaction data consist of the identifiers of the parties, the interaction timestamp and the received signal strength indication (RSSI), a proxy for the distance between devices. This is the data typically captured by contact tracing apps^78^.

**Fig. 7:**
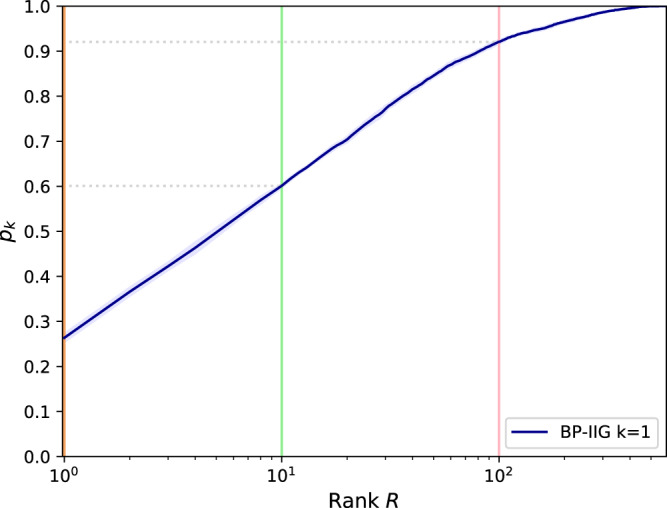
Probability of identification in a bluetooth close-proximity network. We plot *p*_*k*=1_, the probability of identification within rank *R* for *k* = 1. The 95% confidence interval is shown in light blue. Our method correctly identifies people *p*_*k*=1_ = 26.4% of the time based on their 1-IIGs. Out of 10 people (*R* = 10), it is able to identify the right person *p*_*k*=1_ = 60.1% of the time.

Figure 7 shows that for *k* = 1 our approach is able to identify target individuals *p*_*k*=1_ = 26.4% of the time among the 587 people. Out of 10 people (*R* = 10), it is able to identify the right person *p*_*k*=1_ = 60.1% of the time. While our dataset is too small to evaluate for larger values of *k*, we expect the results to further increase when more information is available.

Taken together, our results provide strong evidence of the urgent need to consider profiling attacks when evaluating whether systems, protocols, or datasets satisfy Article 29 WP’s definition of anonymization^31^. In particular, they show how people’s interaction patterns online and offline remain identifiable across long periods of time allowing an attacker to link together data coming from disjoint time periods with high accuracy even in large datasets. Our results challenge current data retention practices and, in the context of the recent COVID-19 pandemic, whether some of the collected data would satisfy the Article 29 linkability criteria. They finally further question the policy relevance of de-identification techniques^86^ and emphasize the need to rethink our approaches to safely use non-personal data. In particular, legal and access control mechanisms are necessary to protect data retained in pseudonymized format, and privacy engineering solutions such as query- and question-and-answer-based systems, local DP mechanisms, or secure-multiparty computation could be deployed to help use data anonymously^87^.

## Discussion

In this paper, we propose a new behavioral profiling attack model exploiting the stability over time of people’s *k*-hop interaction networks. We evaluate its effectiveness on two real-world offline and online interaction datasets and show the risk of identification to be high.

We first compare our attack to previous work from 2014^49^, the only attack in the literature developed for user linkage across call graphs in the context of the D4D challenges (hereafter: ShDa). The method uses a random forest classifier trained on hand-engineered node pair features representative of the nodes’ 2 or 3-hop neighborhoods. The node pair features are pairwise combinations of individual node features consisting of the histogram of each node’s 1-hop or 2-hop neighbors’ degrees. We reimplement their attack for matching nodes from two networks based on nodes’ *k*-hop neighborhood features, *k* ≤ 3, in each network, respectively, and compare their results to ours. For a fair comparison, we convert our attack, which computes a target individual’s match by distance comparison with a list of candidates, into their setup: a binary classifier predicting as positive any pair with distance lower than a threshold (see Supplementary Note 5).

**Fig. 8:**
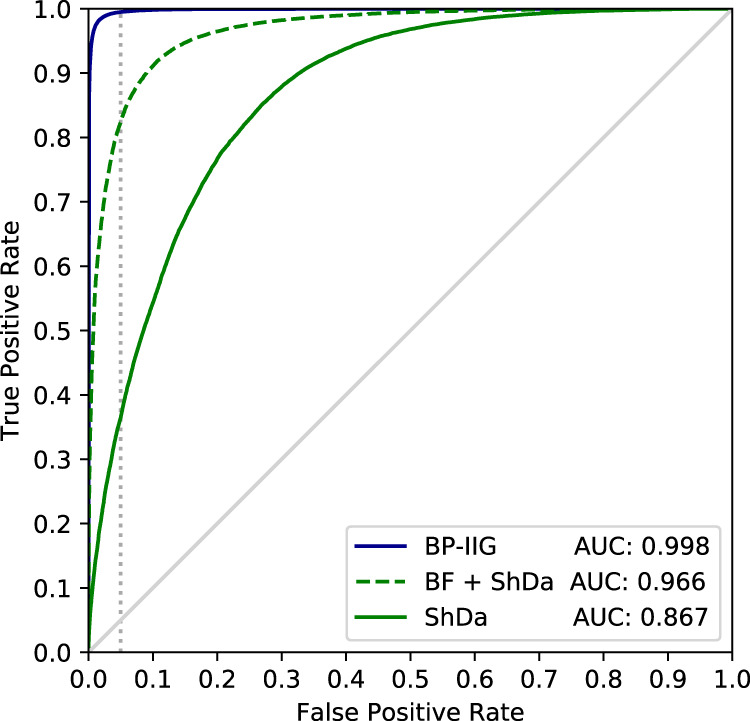
Receiving operator characteristic (ROC) curves with area under the curve (AUC) score for the node pair classification task for *k* = 2. The performance of a random classifier is shown in solid gray. Even for their task, our method (blue line) vastly outperforms both ShDa (solid green line) and its improved version ShDa + BF (dashed green line). For a false positive rate of 0.05, our method achieves a true positive rate of 0.99 vs. 0.36 for ShDa and 0.82 for ShDa + BF (vertical dotted gray line). As shown in Supplementary Fig. 7, both ShDa and ShDa + BF perform poorly when it comes to correctly identifying a person.

Figure 8 shows that our approach (BP-IIG, blue line) vastly outperforms previous work (ShDa, solid green line) making profiling attacks a real risk. We report the receiver operator characteristic (ROC) curve and area under the curve (AUC) on the binary classification task for *k* = 2, showing show our approach achieves, on their task and for a false positive rate of 0.05, a true positive rate of 0.99 (AUC = 0.998) vs. 0.36 for ShDa (AUC = 0.868). Our method still outperforms ShDa when we add to it our behavioral features (ShDA + BF, green dashed line) which result in a true positive rate of 0.82 for a false positive rate of 0.05. We refer the reader to Supplementary Fig. 6 for results for other values of *k*. More importantly, Supplementary Fig. 7 shows how our approaches strongly outperform ShDa on the task of interest: the probability *p*_*k*_ of correctly identifying a person. Here, ShDa alone only achieves a *p*_*k*=2_ = 0.3% versus *p*_*k*=2_ = 52.4% for our attack. Even the improved version, ShDa + BF, only achieves a low *p*_*k*=2_ = 8.3%. Our approach further improves on other baselines (see Supplementary Note 5).

We further validate that our attack generalizes by examining its performances when testing is performed on a set disjoint from the training set in the identities of the individuals, time periods used, or both, as illustrated in Supplementary Fig. 8. Our attack performs similarly across the three scenarios for all values of *k* ∈ {1, 2, 3}, as shown in Supplementary Table 5. Our attack is equally able to identify people unseen during training in time periods also unseen during training (*p*_*k*=2_ = 61.5%) as in cases when the same people (*p*_*k*=2_ = 62.2%) or time periods (*p*_*k*=2_ = 60.5%) used in testing are seen during training. We observe similar results for *k* = 1 and *k* = 3 (see Supplementary Note 6).

While the attack model is general (see Setup), we have throughout the paper assumed that the auxiliary information comes from a time period posterior to the dataset ${{{{{{{\mathcal{D}}}}}}}}$ (${t}_{1}^{\prime} \; < \; {t}_{2}^{\prime}$). Using our BP-IIG (*k* = 2) approach, we compared the performance of a model trained on 9 consecutive weeks of data and tested on the following 9 weeks, with that of a model trained on the last 9 weeks and tested on the first 9 weeks. The two models gave the same performance (*p* = 0.58, see Supplementary Note 7). This confirms the generality of our model.

We here focus on a general attack model which we use to show how both mobile phone and bluetooth interaction data are identifiable across long periods of time. While we do not wish to emphasize specific attack scenarios, examples could include data collectors pseudonymizing interaction data monthly as part of their data retention policy; poorly designed centralized contact tracing apps relying on frequent re-pseudonymization to protect user’s privacy; or the behavioral identification of a phone through e.g. their messaging pattern. The attacker could also be a law enforcement agency with, e.g., the Patriot Act giving intelligence agencies access to the 3-hop graphs of suspects (later restricted to 2-hop under the 2015 USA Freedom Act)^88^.

Our attack model uses a definition of the *k*-IIG that excludes interactions between the *k*-hop neighbors, as already done in the past for mobile call graphs^77^. We consider this to be a realistic assumption e.g. for *k* = 1 when the attacker’s auxiliary information could come from the target’s mobile. In the context of contact tracing, the attacker would have access to the log of the target’s interactions with their contacts but would not have any information on the interactions between its contacts. This assumption makes our results a lower bound of what could be achieved with more information.

While we assume, again in line with previous practices^49,77^, that pseudonyms are identical over time for nodes in *k*-IIGs of the same individual, this is not a requirement of our approach. Re-pseudonymization of nodes over time might be used, for example, to avoid direct access to an individual’s interactions over a long period of time. Our approach would still work even if the dataset ${{{{{{{\mathcal{D}}}}}}}}$ consisted of weekly *k*-IIGs with different pseudonyms for the same node appearing in two weekly *k*-IIGs of the same person, so long as the attacker knows the identity of the originating individual in each weekly *k*-IIG. For *k* = 1, this is due to the approach relying on the originating individual’s behavioral features. For *k* ≥ 2, the graph attention network used is invariant to nodes’ ordering, but the originating individual’s identity is needed for computing the *k*-IIG’s final embedding.

## Methods

### Overview of the attack

We assume that the dataset and the auxiliary data come from disjoint time periods ${{{{{{{{\mathcal{T}}}}}}}}}_{1}$ and ${{{{{{{{\mathcal{T}}}}}}}}}_{2}$, respectively. The attack is based on comparing an individual’s weekly profile extracted from time period ${{{{{{{{\mathcal{T}}}}}}}}}_{2}$ to the weekly profiles of everyone in the dataset, constructed from their respective weekly *k*-IIGs in ${{{{{{{{\mathcal{T}}}}}}}}}_{1}$. The attack thus exploits the weekly patterns in human behavior (e.g., weekdays and weekend). We assume ${{{{{{{{\mathcal{T}}}}}}}}}_{1}$ and ${{{{{{{{\mathcal{T}}}}}}}}}_{2}$ to be at least one week long. The attacker splits the *k*-IIGs from ${{{{{{{\mathcal{D}}}}}}}}=\{{G}_{i,{{{{{{{{\mathcal{T}}}}}}}}}_{1}}^{k}\ \ :\ \ i\in {{{{{{{\mathcal{I}}}}}}}}^{\prime} \}$ by weeks to obtain $\{{G}_{i,{{{{{{{{\mathcal{W}}}}}}}}}_{t}}^{k}:\ \ i\in {{{{{{{\mathcal{I}}}}}}}}^{\prime} ,1\ \le \ t\ \le \ T^{\prime} \}$, where ${{{{{{{{\mathcal{T}}}}}}}}}_{1}={{{{{{{{\mathcal{W}}}}}}}}}_{1}\cup \ldots \cup {{{{{{{{\mathcal{W}}}}}}}}}_{T^{\prime} }$. From ${{{{{{{{\mathcal{T}}}}}}}}}_{2}$, the attacker extracts one target week ${{{{{{{{\mathcal{T}}}}}}}}}_{2}^{\prime}\subset {{{{{{{{\mathcal{T}}}}}}}}}_{2}$ about the target individual *i*_0_. The attacker then singles out the most recent week in ${{{{{{{{\mathcal{T}}}}}}}}}_{1}$, reference week ${{{{{{{{\mathcal{T}}}}}}}}}_{1}^{\prime}\subset {{{{{{{{\mathcal{T}}}}}}}}}_{1}$, to be used for the identification. The remaining data in ${{{{{{{{\mathcal{T}}}}}}}}}_{1}$ are used to train the profiles of *k*-IIGs.

### Preprocessing of a *k*-IIG

The attacker extracts behavioral features at the weekly level, then simplifies each weekly *k*-IIG to a simple graph that can be mapped to an embedding using graph neural networks and optimized for identification.

We use bandicoot^73^, an open-source Python library to compute a set of behavioral features from an individual’s list of interactions. Bandicoot has been used to predict people’s personality^20^, making it a suitable choice for identification. bandicoot takes as input an individual’s list of interactions, consisting of the other party’s unique identifier, the interaction timestamp, type (call or text), direction (in or out), and duration (if a call). The features range from simple aggregated features, e.g., the number of voice and text contacts, to more sophisticated statistics, e.g., the percentage of an individual’s contacts that account for 80% of their interactions. For the Bluetooth close-proximity data, we set the type to call, the direction to out, and the call duration to the negative RSSI. Supplementary Tables 1 and 2 list the features used in this paper for the mobile phone dataset and the Bluetooth close-proximity dataset, respectively.

Using bandicoot, the attacker extracts a set of behavioral features for all nodes in a weekly *k*-IIG with outdegree ≥1 that are at most *k*−1 hops away from the originating individual. In practice, the positive outdegree is a proxy for a node being a subscriber to service *S*. To these features the attacker adds estimates of the percentage of out of network call, texts, call durations, and contacts based on the information available in *k*-IIG. The attacker further removes the featureless nodes from the *k*-IIG and collapses all directed edges between two remaining nodes into a single directed edge of the same direction. The attacker thus simplifies the *k*-IIG ${G}_{i,{{{{{{{\mathcal{T}}}}}}}}}^{k}=(V,E)$ to obtain the simplified *k*-IIG ${\bar{G}}_{i,{{{{{{{\mathcal{T}}}}}}}}}^{k}=(\bar{V},\bar{E})$, a simple graph with $\bar{V}=\left\{v\in V:\ \ v\right.$ is on a path of length at most *k*−1 from node $\left.i\right\}$ ∩ {*v* ∈ *V* : ∃ *w* ∈ *V* with(*v*, *w*, *m*) ∈ *E*} and $\bar{E}=\{e=(v,w)\in \bar{V}\times \bar{V}\ \ :\ \ v\,\ne\, w\wedge \exists (v,w,m)\in E\}$ (see the Supplementary Methods).

### Embedding of *k*-IIG

Our *k*-IIG-based Behavioral Profiling approach (BP-IIG) first computes a time-dependent profile of an individual in the form of a vector representation (embedding) by aggregating the features in ${\bar{G}}_{i,{{{{{{{\mathcal{T}}}}}}}}}^{k}$ using graph neural networks with attention, similarly to the GraphSAGE architecture^66^, but using attention weights^62^, as described in Supplementary Alg. 1. Supplementary Fig. 2B illustrates the model and Supplementary Note 8 shows an analysis of the attention weights. Differently from GraphSAGE, the architecture uses an MLP with a hidden layer instead of a single fully connected layer after each concatenation between the features of the node originating the simplified *k*-IIG and the weighted average of its neighbors’ features. The output of the MLP layer is ${{\mathbb{L}}}_{2}$-normalized.

### Triplet sampling procedure

The embeddings are optimized for identification using the triplet loss^76^ with a triplet sampling procedure designed with the goal of separating the profiles of different individuals in the embedding space. A triplet is composed of an anchor, a positive, and a negative example. The anchor and positive examples are two instances of the same individual, while the negative example is an instance of a different individual. For a given batch size *B*, the triplet sampling procedure works as follows: (1) one week *i* is sampled uniformly at random among the training weeks, (2) *B* individuals are sampled from ${{{{{{{\mathcal{I}}}}}}}}^{\prime}$ and their *k*-IIGs in week *i* are used as anchor examples, while their *k*-IIGs in weeks *i*−1 and *i* + 1 (modulo the number of training weeks) are used as positive examples and 3) for each anchor, a negative example is selected via mini-batch moderate negative sampling^89^. For step 3, all *k*-IIGs in weeks *i*−1, *i* and *i* + 1 coming from the other *B*−1 individuals in the batch are considered as candidate negative examples. In practice, each mini-batch contains 2*B* triplets, because at step 2) two different positive examples are considered for each anchor. Mini-batch gradient descent is used for the optimization. An epoch is defined as a full pass over at least one anchor example of each individual in ${{{{{{{\mathcal{I}}}}}}}}^{\prime}$. As described above, the attacker splits the dataset to obtain $T^{\prime} \times | {{{{{{{\mathcal{I}}}}}}}}^{\prime} | \,$*k*-IIGs as follows: $\{{G}_{i,{{{{{{{{\mathcal{W}}}}}}}}}_{t}}^{k}:i\in {{{{{{{\mathcal{I}}}}}}}}^{\prime} ,1\ \le \ t\ \le \ T^{\prime} \}$, with $T^{\prime}$*k*-IIGs per individual in ${{{{{{{\mathcal{I}}}}}}}}^{\prime}$. Data from $P\ \le \ T^{\prime}$ weeks are used to train the model. There are, therefore, by construction, exactly *P* weekly *k*-IIG instances available for the triplet sampling procedure for each individual in ${{{{{{{\mathcal{I}}}}}}}}^{\prime}$.

### Training setup

In the mobile phone dataset, data from enough weeks are available, so the attacker uses disjoint weeks for training: ${{{{{{{{\mathcal{T}}}}}}}}}_{1}:={{{{{{{{\mathcal{W}}}}}}}}}_{1}\cup \ldots \cup {{{{{{{{\mathcal{W}}}}}}}}}_{T^{\prime} }:={{{{{{{{\mathcal{W}}}}}}}}}_{1:T^{\prime} }$, with ${{{{{{{{\mathcal{W}}}}}}}}}_{1},\ldots ,{{{{{{{{\mathcal{W}}}}}}}}}_{T^{\prime} }$ disjoint and ordered increasingly. Week ${{{{{{{{\mathcal{W}}}}}}}}}_{T^{\prime} }$ is used as reference week ${{{{{{{{\mathcal{T}}}}}}}}}_{1}^{\prime}$ in the attack. For each *k* ∈ {1, 2, 3}, the attacker selects the best hyperparameters using cross-validation on the weeks ${{{{{{{{\mathcal{W}}}}}}}}}_{1:T^{\prime} -1}$, where each test fold is composed of two consecutive weeks. The first week is used as reference week and the auxiliary data about target individuals come from the second week. With $T^{\prime}$ being odd, the $(T^{\prime} -1)/2$ disjoint test folds are defined as $\{({{{{{{{{\mathcal{W}}}}}}}}}_{2i+1},{{{{{{{{\mathcal{W}}}}}}}}}_{2i+2}),0\ \le \ i \; < \; (T^{\prime} -1)/2\}$. For each fold, the previous two time periods (modulo $T^{\prime} -1$) are used as validation weeks for early stopping. The remaining weeks are used for training. Given the best hyperparameter set, the attacker trains the model on data from ${{{{{{{{\mathcal{W}}}}}}}}}_{1:T^{\prime} -3}$, using validation weeks $({{{{{{{{\mathcal{W}}}}}}}}}_{T^{\prime} -2},{{{{{{{{\mathcal{W}}}}}}}}}_{T^{\prime} -1})$ for early stopping. For early stopping, the metric used is *p*_*k*_, the probability of identification within rank 1 on the validation weeks.

In the Bluetooth close-proximity dataset, only 4 weeks, here denoted ${{{{{{{{\mathcal{T}}}}}}}}}_{1}={{{{{{{{\mathcal{W}}}}}}}}}_{1}\cup \ldots \cup {{{{{{{{\mathcal{W}}}}}}}}}_{4}:={{{{{{{{\mathcal{W}}}}}}}}}_{1:4}$ are available. For *k* = 1, the attacker uses the first two weeks of data for training, the second and third week of data for validation, and results are reported on the third and fourth week of data (i.e., ${{{{{{{{\mathcal{T}}}}}}}}}_{1}^{\prime}={{{{{{{{\mathcal{W}}}}}}}}}_{3}$ and ${{{{{{{{\mathcal{T}}}}}}}}}_{2}^{\prime}={{{{{{{{\mathcal{T}}}}}}}}}_{2}={{{{{{{{\mathcal{W}}}}}}}}}_{4}$). In order to increase the number of training samples per individual, the attacker generates 8 overlapping weeks of data from the two training weeks. Because the training data contain a total of 14 days of interactions *d*_1_ ∪ … ∪ *d*_14_, the attacker generates 8 overlapping weeks ${{{{{{{{\mathcal{W}}}}}}}}}_{1}^{\prime},\ldots ,{{{{{{{{\mathcal{W}}}}}}}}}_{8}^{\prime}$, with ${{{{{{{{\mathcal{W}}}}}}}}}_{i}^{\prime}={d}_{i}\cup \ldots \cup {d}_{i+6}$, 1 ≤ *i* ≤ 8.

## Supplementary information

Supplementary Information

## Data Availability

The Bluetooth close-proximity dataset^11^ is available at 10.6084/m9.figshare.7267433. For contractual and privacy reasons, we cannot make the raw mobile phone data available.

## Code availability

To limit the risk of nefarious uses we chose—in coordination with ethics reviewers—to not publicly release the code. We will instead make the code available upon request to the corresponding author to researchers in the field for scientific purposes.

## Impact statement

We hope our findings will help raise awareness of the risk posed by the identifiability of interaction data. In particular, we hope this will encourage the implementation of security measures and the deployment of privacy-preserving systems when collecting, analyzing, and sharing such data.

Our attack is a general profiling attack against interaction data. While we show our attack to be effective against bluetooth interaction data—the same type of data collected by contact tracing applications—we neither attacked nor considered specific applications or protocols. For the avoidance of doubt, we do not believe our results currently apply to robust privacy-preserving contact tracing protocols such as Google and Apple’s Exposure Notification (GAEN).

While the publication of our findings might increase the risk of profiling attacks being used for nefarious purposes, we believe the benefits of these findings being public knowledge, alongside our decision not to release the code and to delete the models upon publication, means that the benefits largely outweigh the risks in general. First, we believe that deploying an attack similar to ours was already possible as the technologies used (graph attention networks, bandicoot features, etc.) are already well-known in the literature. The publication of our results will instead inform practitioners about the risk and enable them to enact security measures. Second, to limit the reach and possible misuse of our attack in practice, we chose—in coordination with ethics reviewers—not to release the code publicly and to only make it available upon request to researchers in the field for scientific purposes. Third, we considered developing and releasing, alongside our findings, technical defenses. While defenses such as noise addition might mitigate the risk, we were not convinced they would effectively prevent future attacks in general. Worse, they might give a false impression that privacy is preserved. Instead, we believe security measures such as access control and privacy-enhancing systems based on provable guarantees to be the best defenses today against profiling attacks.
