# Supplementary material for: Interaction data are identifiable even across long periods of time
Source: Nat Commun. 2022 Jan 25;13:313. doi: 10.1038/s41467-021-27714-6 (PMC8789822; doi:10.1038/s41467-021-27714-6)
Supplement: Supplementary file 1 — Supplementary Information [file 41467_2021_27714_MOESM1_ESM.pdf]

Supplementary Information for "Interaction data are identifiable even across long periods of time"

Ana-Maria Creţu[1,2], Federico Monti[3,4], Stefano Marrone[1,2,5], Xiaowen Dong[6], Michael Bronstein[1,3,4] and Yves-Alexandre de Montjoye[1,2]*

[1] *Department of Computing, Imperial College London, London, SW7 2AZ, UK*
[2] *Data Science Institute, Imperial College London, London, SW7 2AZ, UK*
[3] *Faculty of Informatics, Università della Svizzera Italiana, 6904 Lugano, Switzerland*
[4] *Twitter, London, W1B 5DL, UK*
[5] *University of Naples Federico II, Naples, 80138, Italy*
[6] *Department of Engineering Science, University of Oxford, OX2 6ED, UK*
*To whom correspondence should be addressed; E-mail: demontjoye@imperial.ac.uk

Supplementary Methods

Mobile phone interaction data

Dataset

We use a dataset composed of the 3-IIGs of $N = 43,649$ subscribers of a mobile carrier collected over a period of $T = 35$ consecutive weeks. All N subscribers interacted with at least 5 subscribers in each week. To bound the number of contacts for each person while maintaining as much information as possible, we discard the 3-IIGs with originating individuals having more than 350 subscriber contacts in all of the weeks. The remaining $N = 43,606$ ones interacted with $5 \leq n \leq 350$ different subscribers on each week. The dataset contains a total of 10-100M unique identities and 100M-1B interactions.

Because we want to preserve as many weeks as possible for the increasing time delay experiment while having at least a few training samples for each individual, we set $T' = 15$ for the dataset $\mathcal{D}$.

Simplified k-IIG

As defined in the main paper, $\mathcal{I}$ denotes the set of individuals taking part in the communications recorded by S. Let $\mathcal{I}'' \subseteq \mathcal{I}$ be the set of subscribers to service S. From now on, individuals in $\mathcal{I} \setminus \mathcal{I}''$ will be denoted non-subscribers, or out-of-network individuals. As mobile phone carriers are interoperable, if S is a mobile carrier, then

$\mathcal{I} \setminus \mathcal{I}'' \neq \emptyset$, while for other apps, e.g., a messaging service, $\mathcal{I}'' = \mathcal{I}$. An interaction is described by the two parties v and $w \in \mathcal{I}$, and the interaction data $m = (t, d, t', \delta)$ consisting of the interaction's type t (call or text), the direction d (incoming or outgoing), the timestamp t' (in seconds), and the duration δ (in seconds if a call, or equal to $\perp$ otherwise).

Given two individuals $v, w \in \mathcal{I}''$ and one interaction between the two, the interaction graph $G_{\mathcal{T}}$ contains two directed edges $(v, w, m = (t, d, t', \delta))$ and $(w, v, m' = (t, d', t', \delta))$ with $\{d, d'\} = \{\text{in, out}\}$. The direction (either in or out) is determined by which party initiated the interaction: for example if w calls v, $d = \text{in}$ and $d' = \text{out}$. The other interaction data in m and m' (i.e., the interaction type t, the timestamp t', and the duration δ) are the same.

Given two individuals $v \in \mathcal{I} \setminus \mathcal{I}''$ and $w \in \mathcal{I}''$, only one edge is recorded by S with the subscriber w as source node. For example, if a non-subscriber $v \in \mathcal{I} \setminus \mathcal{I}''$ calls a subscriber $w \in \mathcal{I}''$ at timestamp t' for a duration of δ seconds, $(w, v, m = (\text{call}, \text{in}, t', \delta))$ is recorded but $(v, w, m = (\text{call}, \text{out}, t', \delta))$ is not recorded. We distinguish between individuals in $\mathcal{I} \setminus \mathcal{I}''$ and individuals in $\mathcal{I}''$ because for the latter all their interaction data are available, while for the former only those interactions with subscribers of S are recorded. As a result, for the former, the graph has only incoming edges, corresponding to the interactions with subscribers of S. For the latter, all interactions are recorded and there are both incoming and outgoing edges. When examining a given k-IIG, one does not need to know the subscriber information for a node v (i.e., whether $v \in \mathcal{I}''$ or not), as it can be inferred: if a node v has at least one outgoing edge (v, w, m) recorded by S, we assume they are a subscriber.

The attacker simplifies the k-IIG $G^k_{i,\mathcal{T}} = (V, E)$ (defined in the Methods) to obtain the *simplified k-IIG* $\bar{G}^k_{i,\mathcal{T}} = (\bar{V}, \bar{E})$, a simple graph with node and edge sets $\bar{V}$ and $\bar{E}$ defined as follows:

$$V_1 = \{v \in V \ : \ v \text{ is on a path of length at most } k-1 \text{ from node } i\} \quad (1)$$

$$V_2 = \{v \in V \ : \ \exists w \in V \text{ with } (v, w, m) \in E\} \quad (2)$$

$$\bar{V} = V_1 \cap V_2 \quad (3)$$

$$\bar{E} = \{e = (v, w) \in \bar{V} \times \bar{V} \ : \ v \neq w \wedge \exists (v, w, m) \in E\} \quad (4)$$

Supplementary Fig. 1 shows an example of a simplified 2-IIG. We briefly describe the simplified k-IIG $\bar{G}^k_{i,\mathcal{T}}$'s structure for each k:

- $k = 1$: $\bar{G}^k_{i,\mathcal{T}}$ consists of node i only;
- $k = 2$: $\bar{G}^k_{i,\mathcal{T}}$ consists of node i and all its 1-hop neighbors that are also in $\mathcal{I}''$. There is an edge between two nodes if there exists at least one edge between them in the 2-IIG;

- $k = 3$: $\bar{G}^k_{i,\mathcal{T}}$ consists of node i, all its 1-hop and 2-hop neighbors that are also in $\mathcal{I}''$. There is an edge between two nodes if there exists at least one edge between them in the 3-IIG; For computational reasons, we downsample the set of 2-hop neighbors so as the neighborhood size does not exceed a given threshold τ. To this end, we select the τ nodes with largest number of contacts in the 3-IIG. We fix threshold $\tau = 200$. The contacts are selected according to outgoing edges, which correspond to both incoming and outgoing interactions.

Due to artefacts in the data collection (e.g., missing records), the simplified k-IIG's adjacency matrix is not always symmetric in practice, so we symmetrize it during data preprocessing.

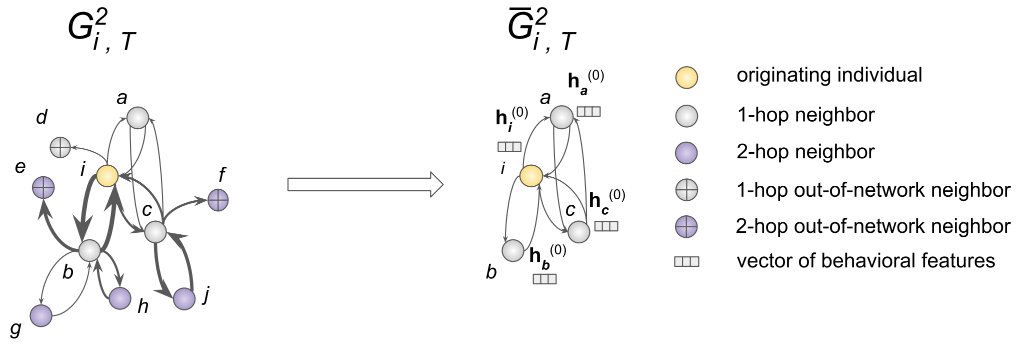

Supplementary Figure 1: An example of a simplified 2-IIG. We show a 2-IIG, $G^2_{i,\mathcal{T}}$ (left), with vertex set consisting of originating individual i (yellow) and its 1-hop (a, b, c and d, gray) and 2-hop (e, f, g, h and j, purple) neighbors, and edges between them displayed as arrows with thickness proportional to the number of interactions. The nodes marked with $+$ (d, e and f) can be considered as out-of-network (i.e., $d, e, f \in \mathcal{I} \setminus \mathcal{I}''$), as they only have incoming edges in the 2-IIG. We further show the simplified 2-IIG $\bar{G}^2_{i,\mathcal{T}}$ (right), consisting of the originating individual i and the 1-hop neighbors, with one edge between any two nodes if there was at least one edge in 2-IIG. In the simplified 2-IIG, all nodes are endowed with behavioral features.

Features

We extract features for all nodes belonging to the vertex sets of weekly simplified k-IIGs. For a week $\mathcal{W}$, let $\bar{G}^k_{i,\mathcal{W}} = (\bar{V}, \bar{E})$ be the simplified k-IIG of originating

individual $i \in \mathcal{I}'$, constructed from k-IIG $G^k_{i,\mathcal{W}} = (V, E)$. By definition, for every $j \in \bar{V}$, j has at least one outgoing edge in the k-IIG and, moreover, all of j's interactions are available in the k-IIG, since j is at most $k-1$ hops away from i, the k-IIGs originating individual.

We use the interactions to generate j's initial feature vector $\mathbf{h}_i^{(0)}$ using the bandicoot toolbox (1). In this work, we chose to focus on the individual behavioral indicators, aggregated over the entire week. Using the `bandicoot.individual.all` method with default parameters [1], we initially extract 33 individual features modelling a user's activity, ranging from simple aggregated features (such as the number of voice and text contacts) to more sophisticated statistics (like the percentage of an individual's contacts that account for 80% of their interactions). Supplementary Table 1 lists the features we keep (1-19) after removing the features with too many 0 or missing values. We removed the features with at least 30% of NULL values, with the exception of the number of incoming texts and the number of text contacts, which can be meaningfully imputed with zeros when the value is missing. We did not keep the number of outgoing texts because it was equal to zero more than 99% of the time when the value was not missing. This was determined using the originating individual's bandicoot features from the first 14 weeks of data.

We further estimate, for each node j in the simplified k-IIG, the value of the percentage of out-of-network calls, contacts, call durations and texts (features 20-23 in Supplementary Table 1) by counting as out-of-network interaction any $(j, l, m) \in E$ such that $\nexists (l, l') \in E$. In other words, we assume l to be a non-subscriber if there is no edge with source l in the k-IIG. Supplementary Fig. 1 shows an example of a 2-IIG having a few nodes that we consider as out-of-network. Although these features are not strictly "behavioral" as the previous ones, and may not apply to a service S to which all parties are subscribers (i.e., when $\mathcal{I}'' = \mathcal{I}$), they can be computed for each k-IIG.

While the bandicoot toolbox is not part of our contribution, for clarity and reproducibility we give below the explicit formulas used to compute each of the features in Supplementary Table 1. We start by introducing useful notations.

As explained previously, the bandicoot features are computed on a weekly basis, only for nodes in a simplified k-IIG, using those nodes' interactions from the k-IIG. For a week $\mathcal{W}$, a k-IIG $G^k_{i,\mathcal{W}} = (V, E)$ and its simplified k-IIG $\bar{G}^k_{i,\mathcal{W}} = (\bar{V}, \bar{E})$, we consider such a node $j \in \bar{V}$. Node j's set of interactions is defined as: $L_j = \{(v, w, m = (t, d, t', \delta)) \in E \ : \ v = j\}$. The interaction data $m = (t, d, t', \delta)$ consists of the interaction's type t (call or text), the direction d (incoming or outgoing), the timestamp t' (in seconds), and the duration δ (in seconds if a call, $\perp$ otherwise). We define the set of contacts as $C_j = \{w \ : \ \exists (v, w, m) \in E \text{ such that } v = j\}$. We will use $\mathbb{I}$ to denote the indicator function and drop the user subscript for the interaction and contact sets: $L := L_j$, $C := C_j$. We define the contact projection function $c : L \to C$ mapping an interaction (v, w, m) to the contact w. Similarly, we define

[1] `https://github.com/computationalprivacy/bandicoot/blob/master/bandicoot/utils.py`

the type, direction, timestamp and call duration projection functions, mapping an interaction $l_1 = (v, w, (t_1, d_1, t'_1, \delta_1)) \in L$ to its type $t(l) = t_1$, direction $d(l) = d_1$, timestamp $t'(l) = t'_1$ and duration $\delta(l) = \delta_1$, respectively.

We further define outofnetwork : $C \to \{\text{True}, \text{False}\}$ mapping a contact w to True if and only if it can be considered as out-of-network (cf. above). We denote by $L_{\text{call}} = \{l \in L \ : \ t(l) = \text{call}\}$ the set of calls and by $L_{\text{text}} = \{l \in L \ : \ t(l) = \text{text}\} = L \setminus L_{\text{call}}$ the set of texts. We denote by $n = |L|$ the number of interactions, by $n_{\text{call}} = |L_{\text{call}}|$ the number of calls and by $n_{\text{text}} = |L_{\text{text}}|$ the number of texts. Last, we denote by $C_{\text{call}} = \{w \in C \ : \ \exists l \in L_{\text{call}} \text{ such that } c(l) = w\}$ the set of call contacts. In what follows, we assume–without loss of generality–that $n_{\text{call}} \geq 1$. Indeed, if this is not the case, a feature involving a division by n_{call}, $|C_{\text{call}}|$, or the total call duration, will be imputed with 0.

1. The percentage of call contacts that account for 80% of the user's total call duration. We denote by $\delta_w = \sum_{l \in L_{\text{call}}} \delta(l)\mathbb{I}(c(l) = w)$ the total call duration between j and another contact $w \in C$. The contacts are ordered decreasingly by δ_w to obtain $\delta_{w_1} \geq \ldots \geq \delta_{w_{|C_{\text{call}}|}}$. Let d the minimum number of contacts such that: $\sum_{1 \leq p \leq d} \delta_{w_p} \geq \lceil 0.8 \cdot (\sum_{1 \leq p \leq |C_{\text{call}}|} \delta_{w_p}) \rceil$. The feature is then defined as $f_1 = d/|C_{\text{call}}|$.

2. The percentage of calls initiated by the user. The feature is defined as $f_2 = (\sum_{l \in L_{\text{call}}} \mathbb{I}(d(l) = \text{out}))/n_{\text{call}}$.

3. The entropy of call contacts. For each contact $w \in C_{\text{call}}$, we denote by $p_w = (\sum_{l \in L_{\text{call}}} \mathbb{I}(c(l) = w))/n_{\text{call}}$ the proportion of call interactions between the user and the contact w. The feature is defined as $f_3 = \sum_{w \in C_{\text{call}}} -p_w \ln p_w$.

4. The percentage of calls that occurred between 7PM and 7AM. Let night : $L \to \{\text{True}, \text{False}\}$ map an interaction l to True if l started between 7PM and 7AM and to False otherwise (given by the timestamp $t'(l)$). The feature is defined as $f_4 = \big(\sum_{l \in L_{\text{call}}} \mathbb{I}(\text{night}(l)\big)/n_{\text{call}}$.

5. The percentage of call contacts that account for 80% of the user's calls. We denote by $n_w = \sum_{l \in L_{\text{call}}} \mathbb{I}(c(l) = w)$ the number of calls between j and another contact $w \in C_{\text{call}}$. The call contacts are ordered decreasingly by n_w to obtain $n_{w_1} \geq \ldots \geq n_{w_{|C_{\text{call}}|}}$. Let d the minimum number of call contacts such that: $\sum_{1 \leq p \leq d} \delta_{w_p} \geq \lceil 0.8 \cdot (\sum_{1 \leq p \leq |C_{\text{call}}|} \delta_{w_p}) \rceil$. The feature is then defined as $f_5 = d/|C_{\text{call}}|$.

6. The number of initiated calls, defined as $f_6 = \sum_{l \in L_{\text{call}}} \mathbb{I}(d(l) = \text{out})$.

7. The number of call contacts, defined as $f_7 = |C_{\text{call}}|$.

8. The number of text contacts, defined as $f_8 = |C_{\text{text}}|$.

9. The number of incoming calls, defined as $f_9 = \sum_{l \in L_{\text{call}}} \mathbb{I}(d(l) = \text{in})$.

10. The number of incoming texts, defined as $f_{10} = \sum_{l \in L_{\text{text}}} \mathbb{I}(d(l) = \text{in})$.

11. The number of days in a week when the user is active. Let day : $L \to \{\text{Monday, Tuesday, Wednesday, Thursday, Friday, Saturday, Sunday}\}$ map an interaction l to the day of the week when it started (given by the timestamp $t'(l)$). The feature is defined as $f_{11} = |\{\text{day}(l) \ : \ l \in L\}|$.

12-13. The mean and standard deviation of times elapsed between two consecutive call interactions. The calls are ordered increasingly by the timestamp to obtain $l_1, \ldots, l_{n_{\text{call}}}$. If $n_{\text{call}} = 1$, the feature cannot be computed (will be imputed with 0 throughout the experiments). If $n_{\text{call}} \geq 2$, the times elapsed between two consecutive calls are computed as $it_p = l_{p+1} - l_p$ for $1 \leq p \leq n_{\text{call}} - 1$. The features f_{12} and f_{13} are defined as the mean and standard deviation of $\{it_p \ : \ 1 \leq p \leq n_{\text{call}} - 1\}$. In particular, $f_{12} = (l_{n_{\text{call}}} - l_1)/(n_{\text{call}} - 1)$.

14-15. The mean and standard deviation of the balance of the user's call contacts. We denote by $b_w = \big(\sum_{l \in L_{\text{call}}} \mathbb{I}(c(l) = w)\mathbb{I}(d(l) = \text{out})\big)/n_{\text{call}}$ the ratio between the number of outgoing calls between user j and contact $w \in C_{\text{call}}$ and the total number of interactions. The features f_{14} and f_{15} are defined as the mean and standard deviation of $\{b_w \ : \ w \in C_{\text{call}}\}$.

16-17. The mean and standard deviation of the number of call interactions with each contact. The features f_{16} and f_{17} are defined as the mean and standard deviation of $\{n_w \ : \ w \in C_{\text{call}}\}$.

18-19. The mean and standard deviation of the call durations. The features f_{18} and f_{19} are defined as the mean and standard deviation, respectively, of durations $\{\delta_l \ : \ l \in L_{\text{call}}\}$.

20. The percentage of out of network call interactions. The feature is defined as $f_{20} = \big(\sum_{l \in L_{\text{call}}} \mathbb{I}(\text{outofnetwork}(c(l))\big)/n_{\text{call}}$.

21. The percentage of out of network contacts. The feature is defined as $f_{21} = \big(\sum_{w \in C} \mathbb{I}(\text{outofnetwork}(w)\big)/n$.

22. The percentage of out of network call duration. The feature is defined as $f_{22} = \big(\sum_{l \in L_{\text{call}}} \delta(l)\mathbb{I}(\text{outofnetwork}(c(l)))\big)/\big(\sum_{l \in L_{\text{call}}} \delta(l)\big)$.

23. The percentage of out of network texts. The feature is defined as $f_{23} = \big(\sum_{l \in L_{\text{text}}} \mathbb{I}(\text{outofnetwork}(c(l)))\big)/n_{\text{text}}$.

During data preprocessing, due to artefacts in the data collection, the simplified k-IIG's adjacency matrix is not always symmetric in practice, so we add edges if needed to make it symmetric.

Feature transformation. We transform the features 6-10 and 16-19 having skewness coefficient ≥ 3 (computed from the N originating individuals' features from the first 14 weeks of data after filling null values with zeroes) by fitting a Box Cox transformation after adding 1 to each feature for it to be strictly positive. In each experiment, the transformation is fitted on data from nodes in the training dataset. The transformation is then applied to training, validation and test nodes.

We then standardize all features to have zero mean and a variance of 1. In each experiment, the mean and standard deviation are estimated from nodes in the training dataset. The estimated values are used to standardize the training, validation and test nodes.

Embedding of a k-IIG. Supplementary Alg. 1 details the feedforward propagation algorithm for computing the embedding of k-IIG, while Supplementary Fig. 2 illustrates it on an example of a 2-IIG. One layer (there are S layers) does one step of propagation on the graph to the neighbors. The embedding of a k-IIG takes as input the simplified k-IIG, $\bar{G}^k_{i,\mathcal{T}} = (\bar{V}, \bar{E})$, where each node $j \in \bar{V}$ is endowed with $\mathbf{h}_j^{(0)}$, the initial vector of behavioral features. For each node j in i's simplified k-IIG, we denote by $\mathcal{N}_{i,k}(j) = \{l \in \bar{V} \ : \ (j,l) \in \bar{E}\}$ their set of neighbors in the simplified k-IIG. Since the node representations are computed independently for each k-IIG, we drop the subscripts for convenience and use $\mathcal{N}(j)$ instead. At each propagation step $s \geq 1$, for each node j in the graph, we compute a new vector representation $\mathbf{h}_j^{(s)}$ by aggregating the features of the node's neighbors $\{\mathbf{h}_l^{(s-1)} \ : \ l \in \mathcal{N}(j)\}$ using graph attention (2) (lines 6-9), then concatenating the obtained representation to the node's features from the previous step $(s-1)$, and last applying a non-linear transformation via a Multilayer Perceptron (MLP, line 10), followed by $\mathbb{L}_2$-normalization (line 11). We use a propagation rule similar to GraphSAGE (3) (line 10) using an MLP with one hidden layer for each step instead of a one-layer fully-connected network.

As an alternative to our attention-based approach, we also implemented a Message Passing Neural Network (MPNN) architecture (4). For the MPNN architecture, the message between a node and a neighbor is computed by applying a linear layer followed by a ReLU nonlinearity to the concatenation of their features. It achieves performance similar to our method (53.5% vs 53.0% on the validation set, $k = 2$, 10 runs, p-value: 0.11, 95% confidence intervals: [52.6, 53.4] for the MPNN and [53.0,

Supplementary Table 1: The list of the 23 features used for the mobile phone interaction data. The bandicoot toolbox methods for computing the features are provided. A dash symbol (-) indicates that the feature was not computed using bandicoot.

	Feature description	Bandicoot method
1	Percentage of call contacts that account for 80% of the total call duration	`percent_pareto_durations`
2	Percentage of initiated calls	`percent_initiated_interactions`
3	Entropy of call contacts	`entropy_of_contacts`
4	Percentage of calls that occurred between 7PM and 7AM	`percent_nocturnal`
5	Percentage of contacts that account for 80% of the user's calls	`percent_pareto_interactions`
6	Number of outgoing calls	`number_of_interactions`
7	Number of call contacts	`number_of_contacts`
8	Number of text contacts	`number_of_contacts`
9	Number of incoming calls	`number_of_interactions`
10	Number of incoming texts	`number_of_interactions`
11	Number of days, between 1 and 7, when the user was active	`active_days`
12-13	Mean/standard deviation of the time between two consecutive calls recorded for the user	`intervent_time`
14-15	Mean/standard deviation of the balance of the user's call contacts, where the balance is the ratio between the number of outgoing calls with a contact and the total number of calls	`balance_of_contacts`
16-17	Mean/standard deviation of the number of calls with each contact	`interactions_per_contacts`
18-19	Mean/standard deviation of call durations	`call_duration`
20	Percentage of out-of-network calls	-
21	Percentage of out-of-network contacts	-
22	Percentage of out-of-network call duration	-
23	Percentage of out-of-network texts	-

53.9] for our approach). We thus opt for the attention-based approach, which is simple and more interpretable.

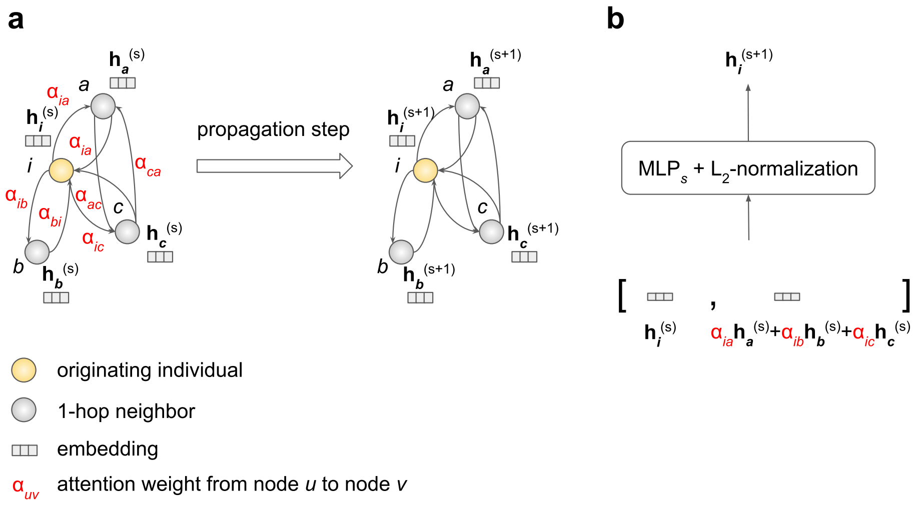

Supplementary Figure 2: One step of propagation to the neighbors on a simplified 2-IIG using graph attention networks. **a** For each node in the 2-IIG, an embedding is computed at step $s+1$ using the embeddings at step s. The attention weights (denoted by α) are computed as shown in Supplementary Alg. 1 (line 7), with superscript symbol s dropped here for illustration purposes. **b** The forward propagation step concatenates a node's embedding at step s to the weighted average of its neighbors' weights, to be given as input to an MLP layer followed by $\mathbb{L}_2$-normalization.

Training setup

We optimize the model parameters of the architecture described in Supplementary Alg. 1 using the triplet loss as explained in the main paper. We use a margin value of $\lambda = 0.25$.

- For $k \in \{2, 3\}$, we use $S = 2$ layers, where MLP_1 has an input size of 46 ($= 2F$), one hidden layer of size 128 and an output size of 50 and MLP_2 has an input size of 100, one hidden layer of size 128 and an output size of 50. For the parameters $\mathbf{v}_s$ and $\mathbf{W}_s$ ($s \in \{1, 2\}$) used to compute the attention weights (line 7 of Supplementary Alg. 1), we use $F' = 20$.
- For $k = 1$, we use a single MLP with input size $F = 23$, a hidden layer of size 128 and output size of 50.

Supplementary Algorithm 1 Forward propagation algorithm for a k-IIG, $k \geq 2$

Inputs:
1. $\bar{G}^k_{i,\mathcal{T}} = (\bar{V}, \bar{E})$, the simplified k-IIG of an individual $i \in \mathcal{I}'$ from time period $\mathcal{T}$
2. Raw features $\mathbf{h}_j^{(0)}$ for each node $j \in \bar{V}$
3. $S \geq 1$, the number of layers. One layer does one step of propagation on the simplified 2-IIG to the neighbors.
4. Learnable Multilayer Perceptron MLP_s, weights $\mathbf{W}_s \in \mathbb{R}^{F' \times F}$ and vector $\mathbf{v}_s \in \mathbb{R}^{2F'}$, for every step $1 \leq s \leq S$

Output:

$\mathbf{h}_i^{(S)}$, the feature vector of node i after the S propagation layers

Initialize:

$\mathbf{h}_j^{(0)} \leftarrow \mathbf{h}_j \in \mathbb{R}^F$, for every node $j \in \bar{V}$

for $s = 1$ to S **do**
  for $j \in \bar{V}$ **do**
    for l such that $(j, l) \in \bar{E}$ **do**
      $\alpha_{jl}^{(s)} \leftarrow \text{LeakyReLU}(\mathbf{v}_s^T[\mathbf{W}_s\mathbf{h}_j, \mathbf{W}_s\mathbf{h}_l])$ # Attention weight for j and l
    end for
    $[\alpha_{j\cdot}^{(s)}] \leftarrow \text{Softmax}([\alpha_{j\cdot}^{(s)}])$
    $\hat{\mathbf{h}}_j^{(s)} \leftarrow \text{MLP}_s[\mathbf{h}_j^{(s-1)}, \sum_{(j,l)\in\bar{E}} \alpha_{jl}^{(s)}\mathbf{h}_l^{(s-1)}]$
    $\mathbf{h}_j^{(s)} \leftarrow \frac{\hat{\mathbf{h}}_j^{(s)}}{\|\hat{\mathbf{h}}_j^{(s)}\|_2}$
  end for
end for

The nonlinearity used is the REctified Linear Unit (RELU).

We train for a maximum number of 100 epochs, decreasing the learning rate by a factor of 2 after 5 epochs of non-increasing probability of identification p_k (i.e., percent of identified individuals) computed over the validation weeks. We stop training if the learning rate decreases below 10^{-5}. We use stochastic gradient descent optimization with a mini-batch size of 64 and a weight decay penalization of 0.001. We perform hyperparameter selection for the learning rate using cross validation as defined in the Methods, with possible values $\eta \in \{0.05, 0.025, 0.01\}$ for $k = 1$ and $\eta \in \{0.01, 0.005, 0.001\}$ for $k = 2, 3$.

Our neural network experiments were carried out in Python 3.7.3 using Pytorch 1.3.1. The random forest approach was executed in Python 3.6.8 using scikit-learn 0.22.1.

Bluetooth close-proximity data

Dataset

We use a Bluetooth close-proximity interaction network of more than 700 university students collected over 4 weeks (5). Interaction data consist of the identifiers of the parties, the interaction timestamp and the Received Signal Strength Indication (RSSI), which could be viewed as a proxy for the distance between devices (5) and is being used in COVID-19 contact tracing apps to measure the risk of infection.

We denote the total time period $\mathcal{T} = \mathcal{W}_{1:4}$ and assume that the attacker has access to (1) an anonymous dataset consists of the 1-IIGs from all participants from the first three weeks $\mathcal{T}_1 = \mathcal{W}_{1:3}$ and (2) auxiliary information about a target individual in the form of a 1-IIG from the fourth week $\mathcal{T}_2 = \mathcal{W}_4$. The attacker uses the first two weeks of data $\mathcal{W}_{1:2}$ for training and the second and third weeks $(\mathcal{W}_2, \mathcal{W}_3)$ as reference, respectively target weeks for validation. Results of the attack will be reported on the third and fourth weeks $(\mathcal{W}_3, \mathcal{W}_4)$, the first of which is used as reference week and the second of which is used as target week.

We use data augmentation in order to increase the number of training samples per individual, as the training dataset only contains two weeks of data. The attacker generates 8 weeks of data from the two training weeks. Because the two training weeks contain a total of 14 days of interactions $d_1 \cup \ldots \cup d_{14}$, the attacker generates 8 overlapping weeks $\mathcal{W}'_1, \ldots, \mathcal{W}'_8$, with $\mathcal{W}'_i = d_i \cup \ldots \cup d_{i+6}$.

We keep all nodes with at least one interaction in all of the weeks: all 8 training weeks derived from $\mathcal{W}_{1:2}$ as well as $\mathcal{W}_3$ and $\mathcal{W}_4$. This results in $N = 587$ people on which results are reported.

Features

Interaction data consists of the identifiers of the parties a and b, the interaction timestamp t and the RSSI r. From one such interaction (a, b, t, r), if a and b are both

users (i.e., they have non-negative identifiers), we will use (a, b, t, r) and (b, a, t, r). If a is a user and b is -1 (empty scan), we will use only $(a, -1, t, r)$. If a is a user and b is -2 (out of study device, from now on denoted out-of-network), we will use only $(a, -2, t, r)$.

Supplementary Table 2 lists the features we compute using the bandicoot tool (features 1-14) (1). To these we add the number of empty scans and percentage of out-of-network interactions. We consider all interactions to be of call type, by replacing the call duration field with $-r$ and by setting the direction to `out`; the direction will not be used and using the call type simply allows us to compute the features using the tool, but doesn't change anything. We use the toolbox's `bandicoot.individual.all` method to compute the features. For clarity and reproducibility, Supplementary Table 2 lists the bandicoot methods used to compute each individual feature. Similar formulas to those provided in Sec. are used by bandicoot to compute the features (where all the interactions are set as calls).

An interaction's timestamp t in the dataset is the number of seconds elapsed since the lowest timestamp (equal to 0). These relative timestamps are multiple of 300 (i.e., 5 minutes). We fix the reference timestamp to be on a Sunday at 00:00 AM, following the authors' suggestion that the dataset started on a Sunday (5). The exact reference timestamp value only impacts one feature: `percent_nocturnal` the percentage of nocturnal interactions (feature 5). We acknowledge that the reference timestamp's exact time of the day might be inaccurate and leave its estimation for future work. Any interaction of the form (a, b, t, r) counts as outgoing for a for computing `balance_of_contacts` the balance of contacts (features 7-8). We transform the features 7-12 having skewness coefficient ≥ 3 (computed from weeks $\mathcal{W}_{1:2}$) by fitting a Box Cox transformation after adding 1 to each feature for it to be strictly positive. The transformation is fitted on data from nodes in the training dataset. The transformation is then applied to training, validation and test nodes.

The features are standardized to have a mean of 0 and a variance equal to 1, where the mean and variance are estimated on the training data.

Training setup

An MLP with one hidden layer of size 64 and output size of 25 is used. The margin value is 0.25. We train for a maximum number of 100 epochs, decreasing the learning rate by a factor of 2 after 5 epochs of non-increasing probability of identification p_k (i.e., percent of identified individuals) computed over the validation weeks. We stop training if the learning rate decreases below 10^{-5}. We use stochastic gradient descent optimization. Hyperparameter search is performed on the batch size $B \in \{16, 32, 64\}$, the learning rate $\eta \in \{0.01, 0.05, 0.1\}$ and the weight decay $\alpha \in \{0.001, 0.01\}$ on the validation data.

Supplementary Table 2: The list of the 16 features used for the Bluetooth close-proximity interaction data. A dash symbol (-) indicates that the feature was not computed using bandicoot.

	Feature description	Bandicoot method
1	Number of days, between 1 and 7, when the user was active	`active_days`
2	Number of contacts	`number_of_contacts`
3-4	Mean/standard deviation of the negative RSSI of the user's interactions	`call_duration`
5	Percentage of interactions that occurred between 7PM and 7AM	`percent_nocturnal`
6	Entropy of contacts	`entropy_of_contacts`
7-8	Mean/standard deviation of the balance of the user's contacts, where the balance is the ratio between the number of outgoing interactions with a contact and the total number of interactions.	`balance_of_contacts`
9-10	Mean/standard deviation of the time between two consecutive interactions recorded for the user	`interevent_time`
11-12	Mean/standard deviation of the number of interactions with each contact	`interactions_per_contacts`
13	Percent of contacts that account for 80% of the user's interactions	`percent_pareto_interactions`
14	Number of interactions	`number_of_interactions`
15	Number of empty scans (i.e., where the other node is -1)	-
16	Percentage of out-of-network calls (i.e., where the other nodes is -2)	-

Supplementary Note 1: Vertex size of simplified k-IIGs

For $k = 2, 3$, we report the mean and standard deviation of the average weekly number of people in the simplified k-IIGs. For each individual among the N people, we compute the average over all of the weeks of the number of people in their simplified k-IIG. The numbers obtained are 17.3 ± 13.4 ($k = 2$), and 121.5 ± 48.8 ($k = 3$).

Supplementary Note 2: Increasing time period length of auxiliary data

To simply evaluate the extent to which more auxiliary data increase accuracy, we combine the predictions from growing sequences of weeks in the auxiliary data, of the form $(\mathcal{W}_{T'+1}, \ldots, \mathcal{W}_{T'+L})_{1 \leq L \leq T-T'}$, where L denotes the number of weeks used as auxiliary data $\mathcal{T}_2$. The predictions are combined using a majority vote, with tie-breaks decided by the lowest total distance between the target individual and the candidate.

We here detail how the predictions are combined. Let $L \in \{1, \ldots, T-T'\}$ and $i \in \mathcal{I}'$ an individual. For $1 \leq l \leq L$, let j_l be the individual whose profile from reference week $\mathcal{W}_{T'}$ is closest to i's profile from target week $\mathcal{W}_{T'+l}$ and let d_{i,j_l} denote the Euclidean distance between the profiles. Let $C = \{j_1, \ldots, j_L\} := \{j'_1, \ldots, j'_c\}$ be the set of distinct identities among the L top-1 candidates. Because top-1 candidates may be the same in different target weeks, $c = |C| \leq L$. For $1 \leq c' \leq c$, we denote by $n_{c'}$ the number of times $j'_{c'}$ is the top-1 candidate and by $d_{c'} = \sum_{j_l = j'_{c'}} d_{i,j_l}$ the sum of distances between i and $j'_{c'}$, where the summation is taken over all l such that $j_l = j'_{c'}$ is the top-1 candidate.

We obtain a new candidate for i using a majority vote, with tie-breaks decided by the lowest total distance between i and the candidate. Using the lexicographic order between pairs of real numbers ($(a,b) \leq (c,d) \Leftrightarrow a < c$ or $a = c$ and $b < d$), this amounts to selecting $j'_{c^*} \in C$ such that:

$$c^* = \underset{1 \leq c' \leq c}{\operatorname{argmin}} (-n_{c'}, d_{c'}) \tag{5}$$

Supplementary Note 3: Analysis of attack performance with increasing population size

To better understand how well the attack scales with the population size, we evaluate its performance when identifying $N' \leq N (N = 43,606)$ people among the same N' people. For this analysis, the final model trained on data from the N people is used. Ten random subsets of size N' are sampled uniformly without replacement and the average probability of identification $p_k(N')$ (defined as the fraction of people among the N' that are correctly identified within rank 1) is computed. The probability of identification $p_k(N')$ is computed for $N' = 2$, then for $N' = 1000$ to $43,000$ using increments of $\Delta N = 1000$, and finally for $N' = N$. The difference quotient is computed as $\Delta p_k(N') = (p_k(N') - p_k(N' - \Delta N'))/\Delta N'$.

Supplementary Fig. 3 shows that the probability of identification decreases with the reference population size, but that $\Delta p_k(N')$ decreases fast as N' grows. The difference quotient seems to be still decreasing around $N' = N$, suggesting that the probability of identification would decrease at an even slower rate for larger values of N'.

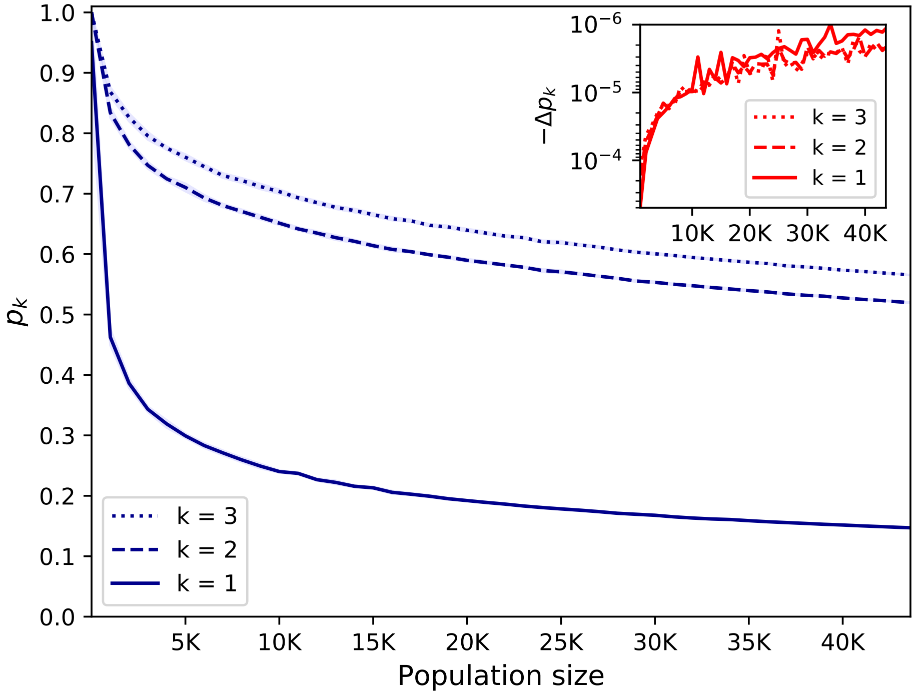

Supplementary Figure 3: Attack's performance with increasing population size. We show $p_k(N')$, the probability of identification within rank 1 for $k \in \{1, 2, 3\}$ in a population of size N'. The 95% confidence interval is shown in light blue. **(Inset)** shows the negative difference quotient $-\Delta p_k(N') = -(p_k(N') - p_k(N' - \Delta(N))/\Delta N'$. The probability of identification decreases with the population size N', but at increasingly slower rates.

Supplementary Note 4: Post-hoc analysis

Comparison with a naïve model

We perform a post-hoc analysis to better understand who are the people correctly identified using our approach. To this end, we compute the fraction of target weeks such that a person is correctly identified by our attack using that week as auxiliary information. We remind the reader that the target weeks which are used as auxiliary information in the attack are the $T'+1,\dots,T$-th weeks in the dataset, and the T'-th week is used as reference week.

The results using our attack are compared with those of a naïve model. This model assumes that individuals are identified independently in each week with the same probability as our approach, and independently from one another. For $l \in \{T'+1,\dots,T\}$, we denote by p_l the probability of identification using our attack with the l-th week as auxiliary information, and by B_l the Bernoulli random variable with probability p_l. Under this model, the number of weeks in which an individual is correctly identified is a random variable $B = \sum_{l=T'+1}^{T} B_l$, with $B_{T'+1},\dots,B_T$ independently distributed. Because the p_l's are not necessarily equal, B follows a Poisson binomial distribution.

We generate N i.i.d. samples of B, then compute the fraction $B/(T-T')$ of target weeks such that a person is correctly identified using that week as auxiliary information, under the naïve model. The results shown in the main paper are computed for one run of our attack and are averaged over 100 repetitions.

We then divide the $N = 43,606$ people in the dataset into two groups based on their identifiability using these results. For each of the 10 runs of our attack ($k = 2$), we choose as cut-off the percentage f of individual weeks where our attack first outperforms the random baseline, thus assigning people to one of two groups: people who are identifiable in at least a fraction f of the weeks, and people who are identifiable in strictly less than a fraction f of the weeks. For all the 10 runs of our attack, the cut-off percentage where our model improves on the baseline is $f = 40\%$. For each individual, we use a majority vote on the labels assigned for the various runs in order to assign a single label.

Feature analysis

To analyse who are the people that our method identifies better than the naïve model, we train a logistic regression to predict the correct label using the features listed in Supplementary Table 3, averaged over the entire set of $T = 35$ weeks. Unless a feature relates to the calls specifically (such as the call duration), the `interaction='callandtext'` argument was used to call the corresponding bandicoot method, else `interaction='call'` was used. Similar formulas to those provided in Sec. are used by bandicoot to compute the features (by using the appropriate interaction type, for example using calls and text instead of calls only for features

14-15).

A logistic regression model is fitted using all the features as predictor variables and the group label as target variable. The resulting coefficients are shown in the main paper. Another logistic regression is fitted for each feature, using it as the sole predictor variable, with the group label as target variable. Supplementary Fig. 4 shows the resulting coefficients, ranked decreasingly by absolute value. Supplementary Table 4 further shows, for each logistic regression, the p-values for the coefficients β (with null hypothesis $\beta = 0$). The `statsmodels` library in Python 3.7.3 was used to fit the models.

In both scenarios (predicting the target variable using all the features vs. each feature individually), the three strongest predictors are the number of interactions, the mean number of interactions per contact and the mean interevent time (i.e., time elapsed between consecutive interactions, see Features). Note that, in the first scenario, increasing the mean interevent time while holding all other features constant over one week amounts to increasing the time span between the first and the last interaction (since this feature is also equal to the time span, divided by the number of interactions). Interestingly, the entropy of contacts, which is ranked towards the bottom when used as sole predictor, is ranked fourth when the other features are accounted for.

Supplementary Fig. 5 reports the Pearson correlation coefficient for each pair of features. We find that the mean of a given summary distribution (e.g., of the number of interactions per contact) is positively correlated with the standard deviation ($\rho > 0.70$). Unsurprisingly, features 17-19 relating to the out-of-network activity are also correlated ($\rho > 0.75$). Interestingly, feature 11 (defined as the mean number of outgoing interactions per contact divided by the total number of interactions, i.e., the total number of outgoing interactions, divided by the product between the number of contacts and the number of interactions) is negatively correlated with the entropy of contacts ($\rho = -0.68$), possibly because in our dataset, people with a more diverse interaction graph (i.e., a larger entropy of contacts) have more incoming activity (normalized by the number of contacts and total number of interactions). Similarly, the number of interactions is negatively correlated with the mean interevent time ($\rho = -0.62$).

Taken together, our results suggest that, when holding all other features constant, either interacting more (features 6 and 13), over a shorter period of time (features 8 or 9), or having a well-balanced interaction graph (features 3, 5 or 14) make someone more identifiable. While our findings suggest the possible influence of the various behavioral features on identification, a causal analysis is beyond the scope of this paper.

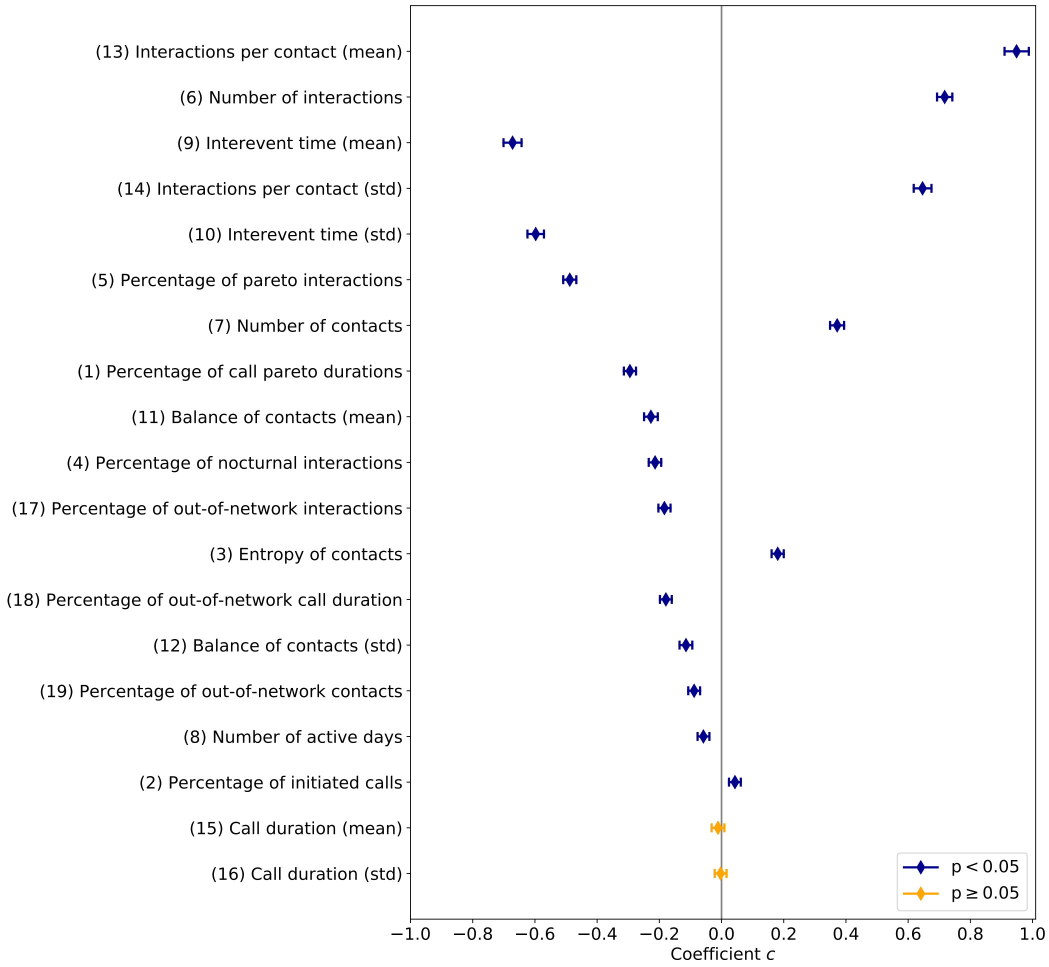

Supplementary Figure 4: Coefficients of a Logistic Regression for individual identifiability using each feature as sole predictor variable. For each feature, we plot the coefficient c (with the 95% confidence interval) of a logistic regression classifier with whether a person is more or less identifiable than expected as target variable, and the feature as predictor variable. Features are ordered decreasingly from top to bottom according to the absolute value of c.

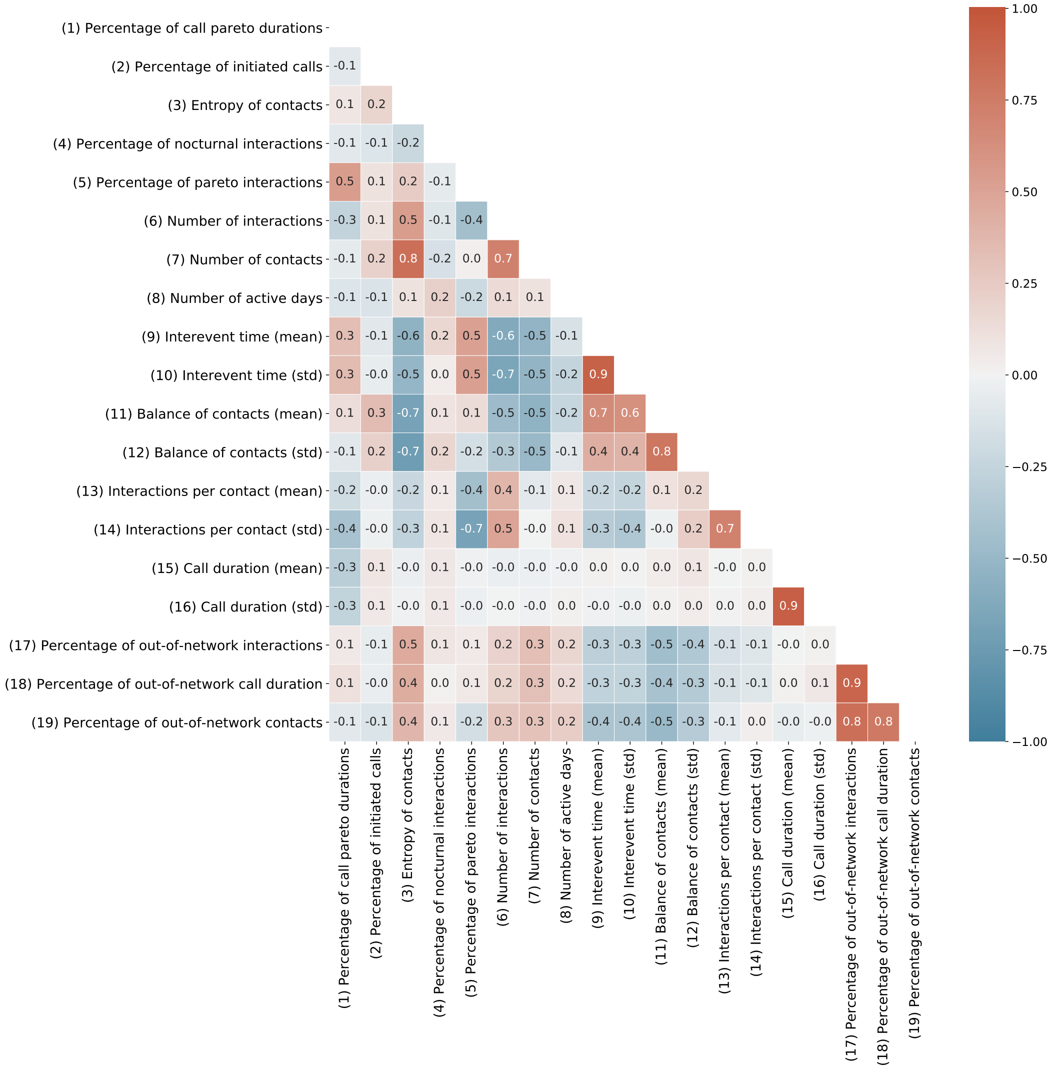

Supplementary Figure 5: Pairwise feature correlations. For each pair of features shown on Supplementary Table 3, we compute the Pearson correlation coefficient. The values displayed are rounded to single decimal precision.

Supplementary Table 3: The list of the 19 features used in the post-hoc analysis. The bandicoot toolbox methods for computing the features are provided. A dash symbol (-) indicates that the feature was not computed using bandicoot.

	Feature description	Bandicoot method
1	Percentage of call contacts that account for 80% of the total call duration	`percent_pareto_durations`
2	Percentage of initiated calls	`percent_pareto_interactions`
3	Entropy of contacts	`entropy_of_contacts`
4	Percentage of interactions that occurred between 7PM and 7AM	`percent_nocturnal`
5	Percentage of contacts that account for 80% of the user's interactions	`percent_pareto_interactions`
6	Number of outgoing calls	`number_of_interactions`
7	Number of contacts (call and text)	`number_of_contacts`
8	Number of days, between 1 and 7, when the user was active	`active_days`
9-10	Mean/standard deviation of the time between two consecutive interactions recorded for the user	`interevent_time`
11-12	Mean/standard deviation of the balance of the user's contacts, where the balance is the ratio between the number of outgoing interactions with a contact and the total number of interactions	`balance_of_contacts`
13-14	Mean/standard deviation of the number of interactions with each contact	`interactions_per_contacts`
15-16	Mean/standard deviation of call durations	`call_duration`
17	Percentage of out-of-network interactions	-
18	Percentage of out-of-network call durations	-
19	Percentage of out-of-network contacts	-

Supplementary Table 4: Logistic Regression summary. For each feature, we report the p-values when all features are used to fit the model **(I)**, respectively when each feature is used as sole predictor variable **(II)**.

	Feature	p-value **(I)**	p-value **(II)**
1	Percentage of call pareto durations	0.000	0.000
2	Percentage of initiated calls	0.000	0.000
3	Entropy of contacts	0.003	0.000
4	Percentage of nocturnal interactions	0.000	0.000
5	Percentage of pareto interactions	0.007	0.000
6	Number of interactions	0.000	0.000
7	Number of contacts	0.017	0.000
8	Number of active days	0.000	0.000
9	Interevent time (mean)	0.000	0.000
10	Interevent time (std)	0.052	0.000
11	Balance of contacts (mean)	0.279	0.000
12	Balance of contacts (std)	0.000	0.000
13	Interactions per contact (mean)	0.000	0.000
14	Interactions per contact (std)	0.001	0.000
15	Call duration (mean)	0.837	0.262
16	Call duration (std)	0.698	0.758
17	Percentage of out-of-network interactions	0.004	0.000
18	Percentage of out-of-network call duration	0.000	0.000
19	Percentage of out-of-network contacts	0.000	0.000

Supplementary Note 5: Comparison with prior work

Approaches considered

We compare our method with what is, to the best of our knowledge, the only attack designed for mobile call k-hop graphs in preparation for Orange's D4D challenge (6).

The method uses a random forest binary classifier trained on hand-engineered node pair features to predict whether they represent the same individual or not. The node pair features are constructed from the nodes' 2 or 3-hop neighborhoods. We implement the same node pair feature-building method and random forest classification algorithm as the approach. For a fair comparison with our method, we further allow the approach to exploit our behavioral features. We thus consider a second approach that uses the same algorithm but where the features of a node pair are the previous features, concatenated to the vector $(|b_i - b'_i|)_{1 \leq i \leq F}$, where $(b_i)_{1 \leq i \leq F}$ and $(b'_i)_{1 \leq i \leq F}$ are two nodes' behavioral features, respectively. We therefore implement 4 baselines: random forest for $k = 2$ and $k = 3$, without (ShDa) or with bandicoot features (ShDa + BF).

For a given node n, the node features used in the attack for a given node are $\mathbf{h}_n^1$, the histogram of the degrees of the node's 1-hop neighbors. For a pair of nodes (n, m) with respective features $(\mathbf{h}_n, \mathbf{h}_m) \in \mathbb{R}^F \times \mathbb{R}^F$, the approach constructs a number of F^2 features of the form $(i, j) \to \frac{|\mathbf{h}_{ni} - \mathbf{h}_{nj}|}{\max(1, \max(\mathbf{h}_{ni}, \mathbf{h}_{nj}))}$ for $1 \leq i \leq F$ and $1 \leq j \leq F$.

We use the out degree (number of contacts) of a node's neighbors in the k-IIG originating at the node. We use $F = 21$ bins: 20 bins of size 5 and one last bin for degrees between 100 and 1000. When the degrees of 2-hop neighbors are available (i.e., for a 3-IIG), we compute an additional feature vector, $\mathbf{h}_n^2$, consisting of the histogram of the degrees of the node's 2-hop neighbors.

We consider seven more approaches for the comparison.

- Two baselines using the histogram of degrees $\mathbf{h}_n^1$ for $k = 2$ with the $\mathbb{L}_2$ distance for the matching ($[\mathbf{h}_n^1, \mathbf{h}_n^2]$ for $k = 3$) (denoted Degree Features (DF) + $\mathbb{L}_2$).
- Two baselines using the histogram of degrees (similarly to the above, for $k = 2$ and $k = 3$, respectively) computed from the number of interactions instead of the number of contacts and the $\mathbb{L}_2$ distance for the matching (denoted All Interaction Features (AIF) + $\mathbb{L}_2$).
- Three baselines using the behavioral features with the $\mathbb{L}_2$ distance for the matching. The first one, to be compared with BP-IIG ($k = 1$), uses all the behavioral features and is denoted BF + $\mathbb{L}_2$. Next, a non graph-based scenario ($k = 0$) where a node's interaction list is available to the attacker, but the contacts' identities are not, is considered. In this scenario, the attacker can exploit an individual's interaction patterns but not the 1-hop graph structure. The baseline proposed uses only the behavioral features that do not exploit the graph information (i.e., features 1-2, 4, 6, 9-13, and 18-19 in Supplementary Table 1, totalling 11 features) (denoted NG-BF + $\mathbb{L}_2$). Third, a baseline

concatenating the behavioral features of a node to those of the top 5 neighbors is considered ($k = 2$). The neighbors are ordered decreasingly by the number of interactions, with tie-breaks decided by the total call duration. We denote this baseline by BF-N + $\mathbb{L}_2$. For each of these baselines, the behavioral features are standardized using estimates from the training weeks (like BP-IIG, our approach).

Experimental setup

For a fair comparison in the node pair binary classification scenario, we define the following pair classifier on any method based on node features (the above five approaches, as well as our method). Given a pair of k-IIGs, we compute the $\mathbb{L}_2$ distance between the embeddings obtained with the method. For every threshold t, a classifier $\mathcal{C}_t$ predicts that a pair of nodes represents the same individual if and only if the distance between the nodes is $\leq t$.

For each baseline, we select the best hyperparameters using 7-fold cross-validation. We use $\frac{T'-1}{2} = 7$ disjoint test folds defined as $\{(\mathcal{W}_{2i+1}, \mathcal{W}_{2i+2}) \ : \ 0 \leq i < 7\}$. In each fold, the remaining data is used for training. We do grid search on the random forest parameters `num_trees` $\in \{100, 400\}$, `max_samples` $\in \{10,000, 100,000\}$ and on using either all the training data or only the two weeks before the test weeks (modulo $T-1$). We add a ninth hyperparameter resembling the most the original settings of the attack (6), with `num_trees` $= 400$, `min_samples_split` $= 0.1$, `max_features` $= 0.05$ and `max_samples` $= 10,000$. Given the best hyperparameter set according to the average accuracy on the test fold (computed using all N positive pairs and N random negative pairs) in $\{(\mathcal{W}_{2i+1}, \mathcal{W}_{2i+2}) \ : \ 0 \leq i < 7\}$, the attacker trains the model on data from $\mathcal{W}_{1:14}$. Test results are reported on $(\mathcal{W}_{15}, \mathcal{W}_{16})$.

We compute the Receiving Operator Characteristic (ROC) curve with Area Under the Curve (AUC) score for the node pair classification task. For the evaluation, we consider for every person one positive pair (the same person in weeks $\mathcal{W}_{15}$ and $\mathcal{W}_{16}$) and one negative pair (another person sampled uniformly at random in $\mathcal{W}_{15}$ and the same person in $\mathcal{W}_{16}$). A pair's score is the probability of the pair representing the same individual for the random forest approach or $\frac{1}{1+||e(a)-e(b)||_2}$, where $e : a \to e(a)$ denotes an embedding methods.

Results

Supplementary Fig. 6 shows that our method outperforms all other approaches in the node pair classification scenario. For $k \in \{2, 3\}$, our method clearly outperforms the random forest method (ShDa) (6), which might be explained by the fact that the latter's features rely on the graph structure only. However, the latter is still outperformed by our approach, even when exploiting the behavioral features (ShDa + BF). The nearest neighbor approaches using the Degree Features (DF + $\mathbb{L}_2$) and All Interaction Features (AIF + $\mathbb{L}_2$) perform comparably for $k = 2$ and $k = 3$. Both

approaches are inferior to the learning approach using random forests according to the AUC score, which might be expected since they are not using learning. The approach using the concatenated behavioral features of the neighbors (BF-N + $\mathbb{L}_2$) is inferior to all of the other approaches, including BF + $\mathbb{L}_2$,(whch uses a subset of its features), possibly due to the noise coming from the ordering of the neighbors. When $k = 1$, our BP-IIG method outperforms BF + $\mathbb{L}_2$, the approach using behavioral features. The latter is superior to NG-BF + $\mathbb{L}_2$ ($k = 0$), the baseline which does not use the graph-based behavioral features, suggesting that the graph information is useful for the identification even in the 1-hop case.

Supplementary Fig. 7 shows that our method ($k \in \{1, 2, 3\}$) vastly outperforms all the other approaches in the identification scenario. When $k = 2$ and 3, all baselines using only the graph structure, i.e., ShDa, AIF + $\mathbb{L}_2$ and DF + $\mathbb{L}_2$, perform very poorly in this scenario, with a probability of identification within rank 1 of less than 1%. When we augment the random forest method with the bandicoot behavioral features, its performance increases to 8.3% for $k = 2$ and 9.6% for $k = 3$, suggesting that the behavioral features are helping. When $k = 1$, our method performs better, at 14.7%, than the BF + $\mathbb{L}_2$ baseline at 7.9%, which in turn outperforms the non-graph behavioral features-based approach, NG-BF + $\mathbb{L}_2$ ($k = 0$), which only achieves 2.7%. Interestingly, in the identification scenario, the approach using the concatenated behavioral features of the neighbors (BF-N + $\mathbb{L}_2$) is superior to most baselines for the lower rank values, unlike in the node pair classification scenario. Notably, it achieves a probability of identification within rank 1 of 5.8%, comparable to, albeit lower than BF + $\mathbb{L}_2$ at 7.9%.

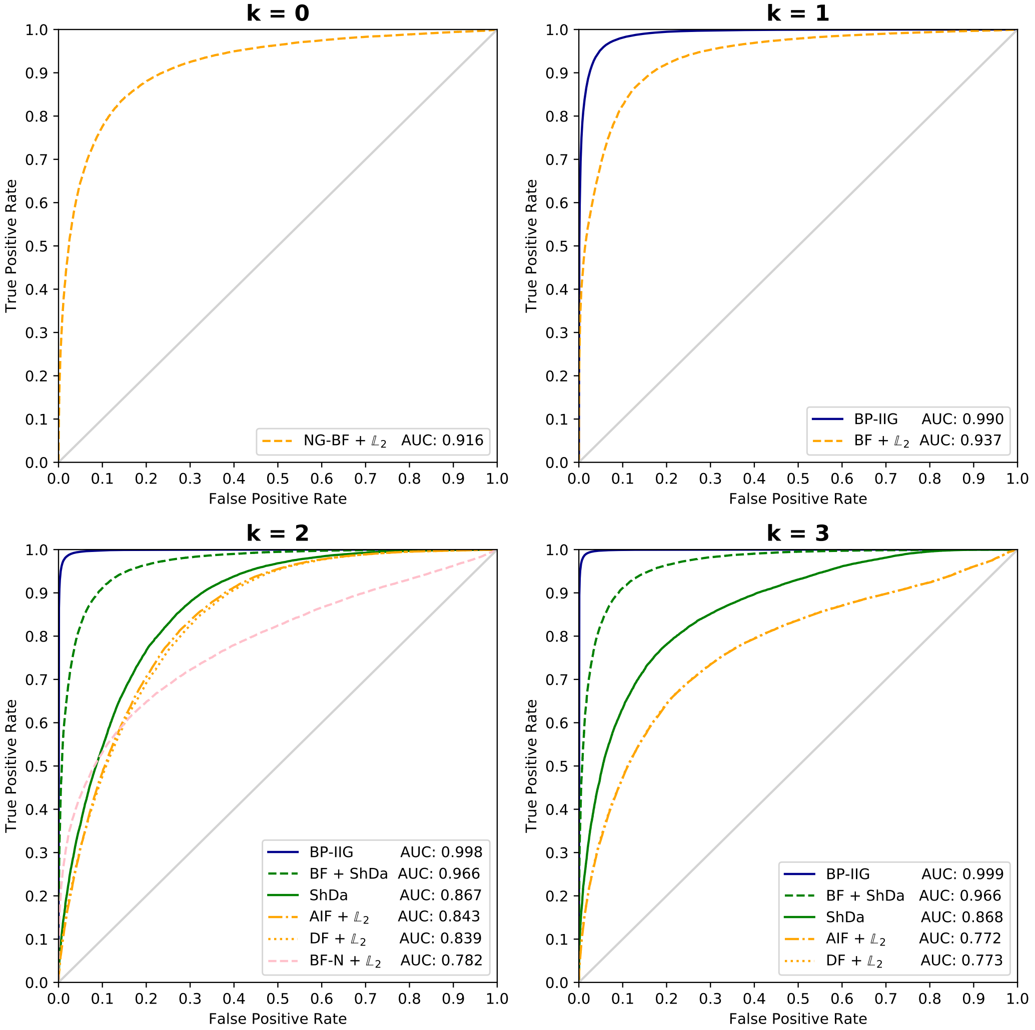

Supplementary Figure 6: Receiving Operator Characteristic (ROC) curve with Area Under the Curve (AUC) score for the node pair classification task. The performance of a random classifier is shown in solid gray. For each $k \in \{1, 2, 3\}$, our method outperforms the baselines.

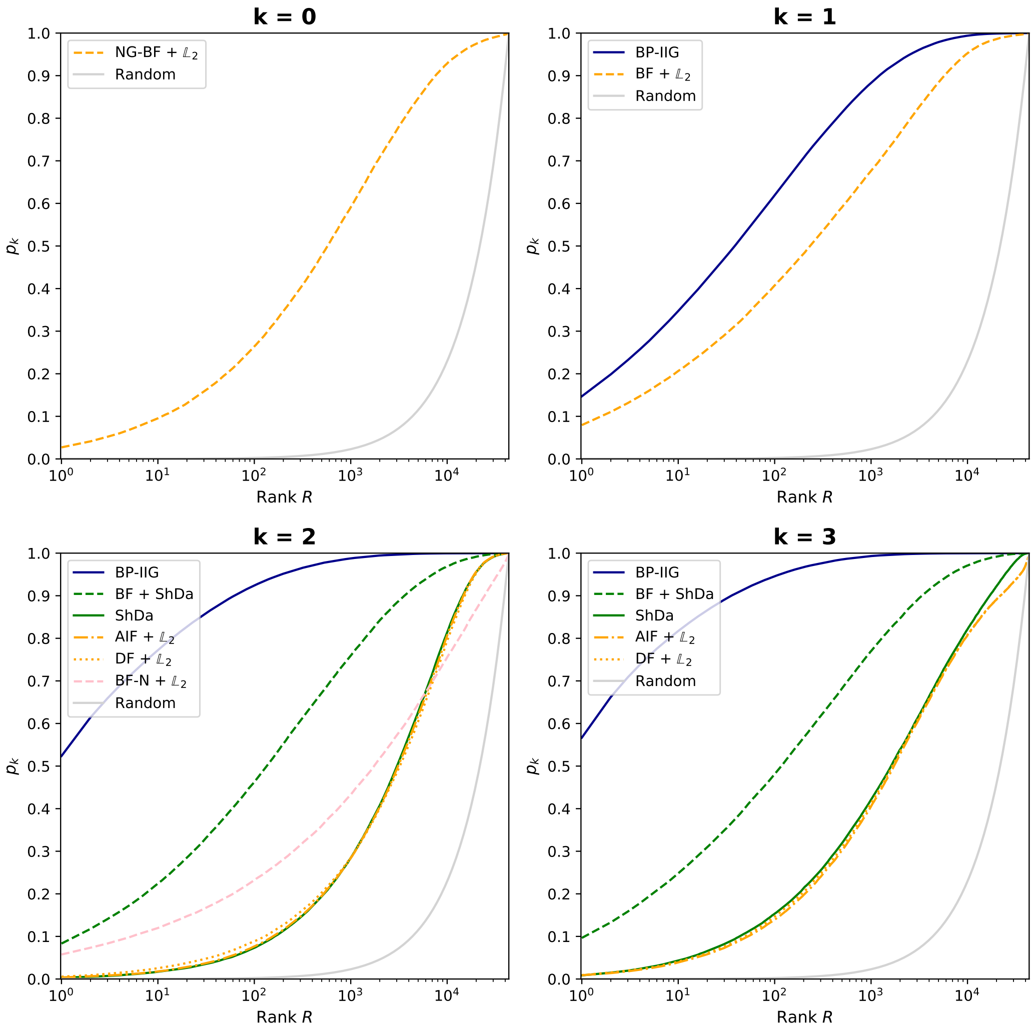

Supplementary Figure 7: p_k, the probability of identification within rank R when the time gap is one week, $R \in \{1, \ldots, N\}$. For each $k \in \{1, 2, 3\}$, our method outperforms all the other approaches.

Supplementary Note 6: Generalization to other identification scenarios

In this section, we study the robustness of our attack when testing is performed on a set disjoint from the training set in the identities of the individuals, time periods used, or both. We use a dataset composed of weeks 1 to $T' + 1 = 16$ and all k-IIGs to design the following experiment. Supplementary Fig. 8 illustrates the three scenarios:

1. the testing, validation and training sets are disjoint in the time periods (Supplementary Fig. 8a and 8b). This is the scenario considered in the main paper, with the data split as in Supplementary Fig. 8a.

2. the testing, validation and training sets are disjoint in the identities of the originating individuals of the k-IIGs (Supplementary Fig. 8c). This scenario might be transductive, i.e., some people's features used in test might have been seen during training. Indeed, in this scenario there might be partial overlap between the k-IIGs because the model is tested on data from weeks seen during training. For example, Alice, the originating individual for a k-IIG in the training set, could be a neighbor of Bob, the originating individual for a k-IIG in the testing set.

3. the testing, validation and training sets are disjoint in the time periods and the identities of the originating individual of the k-IIGs (Supplementary Fig. 8d).

For a fair comparison in the current experiment, the first scenario (Supplementary Fig. 8b) uses the first $T' - 3$ weeks of data from half the k-IIGs for training, while the other two scenarios are trained on the other half of the users and all weeks (Supplementary Fig. 8c) or weeks 1 to $T' - 3$ (Supplementary Fig. 8d) respectively. The validation and test sets are the same across the three scenarios. In the main experiment reported in the paper, we used the first scenario with training on all k-IIGs (Supplementary Fig. 8a). For this experiment, for each $k \in \{1, 2, 3\}$ and each of the three scenarios, we select the best learning rate on the validation set and report results on the test set. The possible values for the learning rate are, as before, $\eta \in \{0.05, 0.025, 0.01\}$ for $k = 1$ and $\eta \in \{0.01, 0.005, 0.001\}$ for $k = 2, 3$.

Supplementary Table 5 shows that our attack performs similarly across the three scenarios. Results suggests that the attack is robust, by being able to identify people unseen during training in time periods also unseen during training (Supplementary Fig. 8d) on par with scenarios when the same people used in testing are seen during training (Supplementary Fig. 8b) or data from the same period is seen during training (Supplementary Fig. 8c).

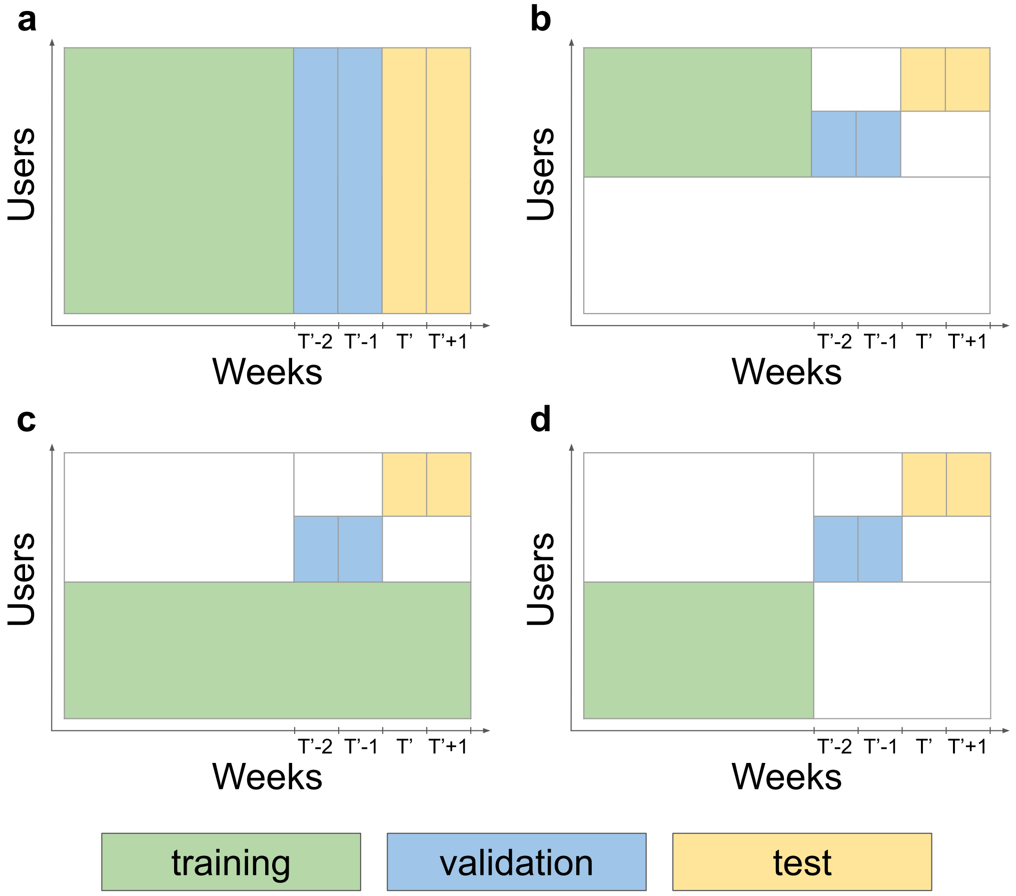

Supplementary Figure 8: The various evaluation scenarios. Testing, validation and training are performed on sets disjoint in the time periods (**a** and **b**), the identities of the originating individuals of the k-IIGs (**c**) or the time periods and the identities of the originating individuals of the k-IIGs (**d**). The green, blue and yellow dataset parts are used for training, validation and testing, respectively. In the validation and test parts, the first week is used as reference week and the second one as target week. Scenario **a** is used for the main experiment of the paper, while the others are used to evaluate the generalization ability of our attack.

Supplementary Table 5: The probability of identification p_k within rank 1 computed for individuals in the test set when compared with users from the reference week, when the time delay is one week for the three scenarios comparison, $k \in \{1, 2, 3\}$. By design, the test set is common across the three scenarios.

	Split by week (%)	Split by individuals (%)	Split by individuals and weeks (%)
$k = 1$	22.8	23.1	23.0
$k = 2$	61.5	62.2	60.5
$k = 3$	66.5	66.8	66.6

Supplementary Note 7: Robustness to training on future data

While the attack model is general, we have throughout the paper assumed that the auxiliary information comes from a time period posterior to the dataset $\mathcal{D}$ ($t'_1 < t'_2$). Using our BP-IIG ($k = 2$) approach, we compared the performance of a model M_1 trained on 9 consecutive weeks of data and tested on the following 9 weeks, with that of a model M_2 trained on the last 9 weeks and tested on the first 9 weeks.

Using $k = 2$ and weeks 18-26 and 27-35, respectively, we found that M_1's and M_2's performances averaged over the 9 weeks that were not used in their training are 36.9% (5.8) and 38.5% (4.7), respectively. The confidence intervals at 95% are [31.7, 42.1] and [34.3, 42.8], respectively. A Welch's t-test results in a p-value of 0.58, therefore not allowing the null hypothesis (that the two means are equal) to be rejected.

Supplementary Note 8: Analysis of attention weights

We perform an analysis of the attention weights to gain insights into the graph neural network's capacity to assign different weights to different neighbors of a node in the aggregation step.

We consider the $N = 43,606$ originating individuals together with their 2-IIGs in the T'-th week, the reference week for the attack used throughout this paper. We remind the reader that for every individual i with neighbor set $\mathcal{N}(i)$ in i's simplified k-IIG (see Supplementary Alg. 1 for more details), for every propagation step $1 \leq s \leq S = 2$, the graph neural network computes a set of attention weights $\{\alpha_{ij}^{(s)} : j \in \mathcal{N}(i)\}$. An intermediate representation $\sum_{j \in \mathcal{N}(i)} \alpha_{ij}^{(s)} \mathbf{h}_j^{(s-1)}$ is used to compute a new representation of node i, where $\mathbf{h}_j^{(s-1)}$ denotes neighbor j's representation from the previous step. The attention weights are computed as a non-linear parametrized function of $\mathbf{h}_i^{(s-1)}$ and each neighbor $\mathbf{h}_j^{(s-1)}$, are positive, and normalized to sum to 1 over the neighbors.

Supplementary Fig. 9a shows the cumulative distribution of the normalized entropy of the attention weight vectors. Supplementary Table 6 shows three random samples for various entropy intervals. The normalized entropy of a vector $(w_1, \ldots, w_{|\mathcal{N}(i)|})$ is defined as the ratio between the entropy $\sum_{1 \leq q \leq |\mathcal{N}(i)|} -w_q \ln(w_q)$ of the vector and $\ln(|\mathcal{N}(i)|)$, the maximum entropy for a discrete probability distribution with $|\mathcal{N}(i)|$ possible values. This shows that while entropy values tend to be high there is sufficient and meaningful variation in the attention weights, which suggests that the model is able to capture the relative importance of neighbors. This is further supported by Supplementary Fig. 9b which shows the cumulative distribution of the normalized range of a vector, defined as $\mathbf{w} \to (\max(\mathbf{w}) - \min(\mathbf{w}))/\min(\mathbf{w})$. In more than 90% of the cases, $\max(\mathbf{w}) - \min(\mathbf{w}) \geq \min(\mathbf{w})$, i.e., $\max(\mathbf{w}) \geq 2\min(\mathbf{w})$ which means that the largest attention weight is at least twice as large as the smallest one(s). This further shows how attention weights help discriminate between the neighbors.

Supplementary Table 6: Examples of attention weight vectors for various intervals of the normalized entropy. The examples are sampled uniformly at random from the given interval for the first propagation step ($s = 1$). In each example, the weights are sorted decreasingly. We can see that in all cases, one or two neighbors have an attention weight at least twice as large as the lowest attention weights.

Interval	Normalized entropy	Attention weight vector
[0.85, 0.90)	0.8990	[0.26, 0.24, 0.19, 0.17, 0.06, 0.04, 0.04]
[0.90, 0.95)	0.9495	[0.26, 0.19, 0.11, 0.10, 0.09, 0.09, 0.08, 0.08]
[0.95, 1.00]	0.9827	[0.24, 0.14, 0.13, 0.13, 0.12, 0.12, 0.12]

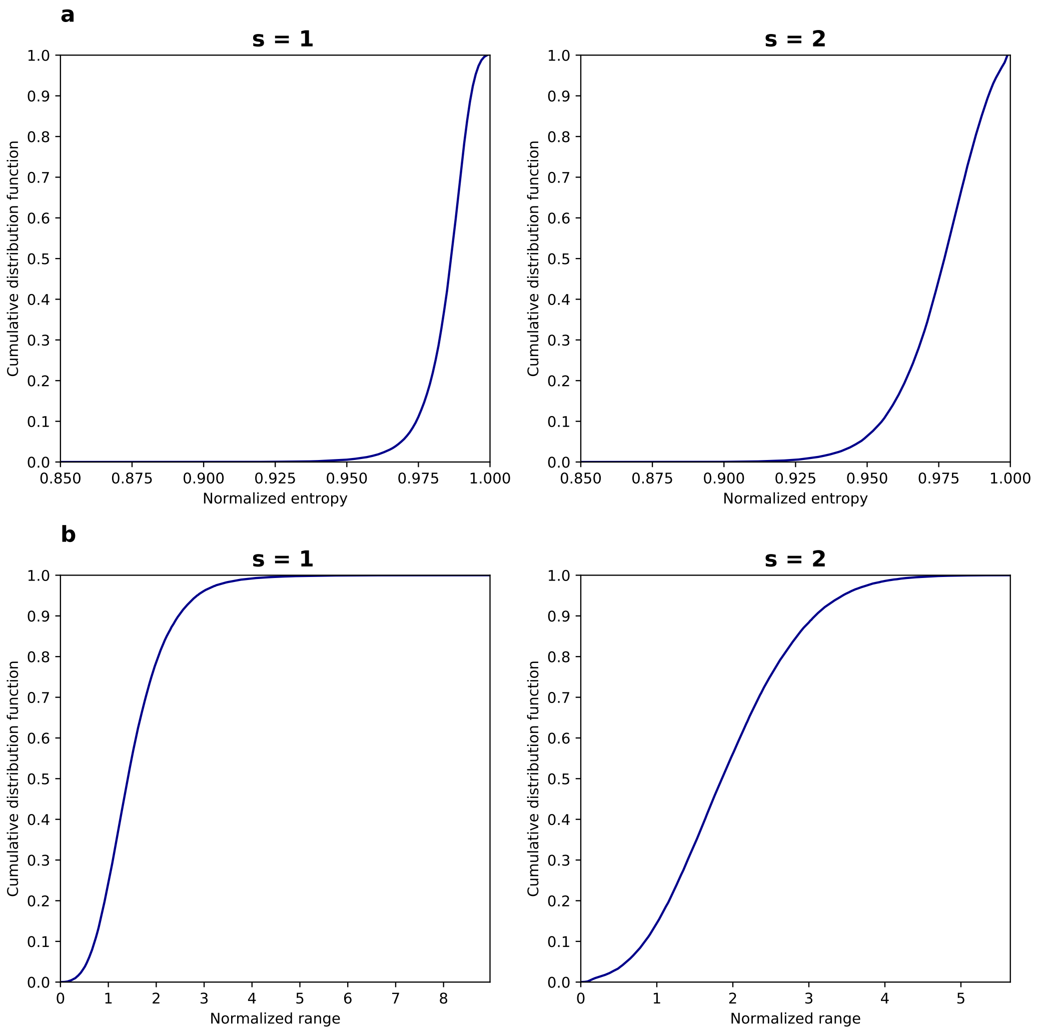

Supplementary Figure 9: Normalized entropy and normalized range for the attention weights. For each originating individual in the dataset ($N = 43,606$), the cumulative distribution function of the normalized entropy **a** and range **b** of the attention weights used to aggregate their neighbors are shown. Two propagation steps are used, each column showing the corresponding attention weights.

Supplementary References

[1] de Montjoye, Y.-A., Rocher, L. & Pentland, A. S. bandicoot: A python toolbox for mobile phone metadata. *The Journal of Machine Learning Research* **17**, 6100–6104 (2016).

[2] Veličković, P. *et al.* Graph attention networks. In *International Conference on Learning Representations* (2018).

[3] Hamilton, W., Ying, Z. & Leskovec, J. Inductive representation learning on large graphs. In *Advances in neural information processing systems*, 1024–1034 (2017).

[4] Gilmer, J., Schoenholz, S. S., Riley, P. F., Vinyals, O. & Dahl, G. E. Neural message passing for quantum chemistry. In *Proceedings of the 34th International Conference on Machine Learning-Volume 70*, 1263–1272 (2017).

[5] Sapiezynski, P., Stopczynski, A., Lassen, D. D. & Lehmann, S. Interaction data from the Copenhagen Networks Study. *Scientific Data* **6**, 1–10 (2019).

[6] Sharad, K. & Danezis, G. An automated social graph de-anonymization technique. In *Proceedings of the 13th Workshop on Privacy in the Electronic Society*, 47–58 (2014).
